# A Bayesian Account of Psychopathy: A Model of Lacks Remorse and Self-Aggrandizing

**DOI:** 10.1162/cpsy_a_00016

**Published:** 2018-10

**Authors:** Aaron Prosser, Karl J. Friston, Nathan Bakker, Thomas Parr

**Affiliations:** Michael G. DeGroote School of Medicine, McMaster University, Hamilton, Canada; Wellcome Trust Centre for Neuroimaging, Institute of Neurology, University College London, London, UK; Department of Psychiatry, University of Toronto, Toronto, Canada; Wellcome Trust Centre for Neuroimaging, Institute of Neurology, University College London, London, UK

**Keywords:** psychopathy, psychopathic personality disorder, antisocial personality disorder, personality disorders, active inference, Bayesian brain, predictive coding, free-energy

## Abstract

This article proposes a formal model that integrates cognitive and psychodynamic psychotherapeutic models of psychopathy to show how two major psychopathic traits called *lacks remorse* and *self-aggrandizing* can be understood as a form of abnormal Bayesian inference about the self. This model draws on the predictive coding (i.e., active inference) framework, a neurobiologically plausible explanatory framework for message passing in the brain that is formalized in terms of hierarchical Bayesian inference. In summary, this model proposes that these two cardinal psychopathic traits reflect entrenched maladaptive Bayesian inferences about the self, which defend against the experience of deep-seated, self-related negative emotions, specifically shame and worthlessness. Support for the model in extant research on the neurobiology of psychopathy and quantitative simulations are provided. Finally, we offer a preliminary overview of a novel treatment for psychopathy that rests on our Bayesian formulation.

## INTRODUCTION

Patients with psychopathic personality traits are clinically complex and challenging to treat. Although there is growing evidence that psychopathic traits can improve, albeit modestly, over time throughout adulthood (Bergstrøm, Forth, & Farrington, 2015; Black, Baumgard, & Bell, 1995; Harpur & Hare, 1994; Ullrich & Coid, 2009), robust evidence for effective psychotherapeutic or pharmacological treatments remains elusive (D’Silva, Duggan, & McCarthy, 2004; Gibbon et al., 2010; Harris & Rice, 2006; Khalifa et al., 2010; Polaschek & Daly, 2013; Reidy, Kearns, & DeGue, 2013; Salekin, 2002; Salekin, Worley, & Grimes, 2010). Moreover, psychopathy is a chronic disturbance, with stable trajectories across developmental periods and origins in externalizing problems (e.g., conduct disorder) and temperamental disturbances (e.g., callous/unemotional traits) in childhood and adolescence (Black, 2015; Frick, Ray, Thornton, & Kahn, 2014; Hare, Neumann, & Widiger, 2012; Moffitt, 1993). In addition to this stability, another factor that makes psychopathy a treatment challenge is that it is one of the strongest predictors of violent and criminal behavior (Andrews & Bonta, 2010a, 2010b; Bonta, Blais, & Wilson, 2014; Bonta, Law, & Hanson, 1998; Gendreau, Little, & Goggin, 1996; Leistico, Salekin, DeCoster, & Rogers, 2008; R. Yu, Geddes, & Fazel, 2012). This means that patients with this personality disorder require complex management strategies to ensure the safety of clinical teams and the public, while facilitating patient rehabilitation and recovery.

These clinical challenges are compounded by the fact that psychopathy is associated with an immense socioeconomic burden and high prevalence. Estimates of the societal costs of psychopathy far exceed the annual costs of alcohol/substance abuse, obesity, smoking, and schizophrenia (Kiehl & Hoffman, 2011). Psychopathy affects approximately 1% of the general population (Coid, Yang, Ullrich, Roberts, & Hare, 2009; Neumann & Hare, 2008; Torgersen, 2012), which is comparable to schizophrenia (Messias, Chen, & Eaton, 2007). Approximately 4%–8% of the psychiatric population and 15%–25% of the correctional population are affected by psychopathy (Hare, 2003; Skeem & Mulvey, 2001; Torgersen, 2012). The high prevalence and substantial socioeconomic burden mean that understanding the etiology of psychopathy is a pressing scientific priority.

This article offers a formal explanation of the pathogenesis of two major psychopathic personality traits—*lacks remorse* and *self-aggrandizing*—in terms of active Bayesian inference. Our formulation draws on one of the most influential neurobiologically plausible explanatory frameworks for message passing in the brain: *predictive coding*. Predictive coding treats the brain as a hierarchical Bayesian inference machine (Friston, 2010; Friston, Stephan, Montague, & Dolan, 2014). This article thus builds on the growing recognition of predictive coding as the framework for understanding the etiology of various psychopathologies (Corlett & Fletcher, 2014; Friston, Stephan et al., 2014; Montague, Dolan, Friston, & Dayan, 2012; Prosser, Helfer, & Leucht, 2016). The predictive coding framework has provided neurobiologically plausible computational models of the etiology of delusions, hallucinations, functional (“hysterical”) symptoms, depression, and autism in terms of abnormal Bayesian inferences (Adams, Stephan, Brown, Frith, & Friston, 2013; Chekroud, 2015; Edwards, Adams, Brown, Pareés, & Friston, 2012; Lawson, Rees, & Friston, 2014; Pellicano & Burr, 2012). This work holds promise to help clinicians and researchers understand the etiology—and thus treatment—of these major psychopathologies. Although inference about oneself has been considered (Moutoussis, Fearon, El-Deredy, Dolan, & Friston, 2014), to date, there has be no formal application of predictive coding to understanding the etiology of psychopathy.

The first section of the article reviews the construct of psychopathy and justifies our focus on these two key traits. The second section reviews the commonalities between cognitive and psychodynamic etiological models of psychopathy from the psychotherapeutic literature. We then describe a Bayesian model of psychopathy and provide quantitative simulations of this model. Furthermore, we show how this model is supported by research on the neurobiology of psychopathy. In the final section, we outline potential treatment implications of the Bayesian model. Our framework integrates cognitive and psychodynamic psychotherapeutic models to show how *lacks remorse* and *self-aggrandizing* can be modeled as a form of abnormal Bayesian inference leading to false beliefs about the self. In brief, these traits reflect entrenched maladaptive Bayesian inferences about the self, which defend against the conscious experience of deep-seated self-related negative emotions, specifically, shame and worthlessness.

## THE CONSTRUCT OF PSYCHOPATHY

Before providing an operational definition of psychopathy, we first need be clear about what is meant by a *personality disorder* (PD), based on the current empirical understanding of the nature and structure of PD, because psychopathy is a particular kind of PD. The general criteria for PD in Section II of the *Diagnostic and Statistical Manual of Mental Disorders*, 5th edition (*DSM–5*; American Psychiatric Association [APA], 2013) retains the original *DSM–IV* definition (APA, 1994). It defines PD as an enduring, pervasive, and inflexible pattern of inner experience and behavior that deviates markedly from the expectations of the individual’s culture (APA, 2013). This pattern of disturbance is manifested in ways the patient perceives and interprets their self, others, and events; the range, intensity, lability, and appropriateness of their emotional responses; and their interpersonal functioning and impulse control. Furthermore, a PD leads to clinically significant distress or impairment and has a stable and long duration whose onset can be traced back to at least adolescence or early adulthood. Furthermore, because the *DSM–5* Section II PD model is a categorical diagnostic system, it classifies personality pathology into 10 distinct disorders grouped into three clusters: Cluster A (paranoid, schizoid, schizotypal), Cluster B (antisocial, borderline, histrionic, narcissistic), and Cluster C (avoidant, dependent, obsessive-compulsive).

The *DSM–5* Section II general criteria of PD have been extensively criticized over the years because they suffer from significant conceptual and empirical problems (Livesley, 1998; Livesley & Jang, 2000; Morey, Bender, & Skodol, 2013; Morey et al., 2011; Parker et al., 2002; Skodol, Bender et al., 2011; Skodol, Clark et al., 2011). First, these criteria are nonspecific to PD, because many other mental disorders meet some or all of the criteria. Second, there is no empirical basis for these general criteria. Furthermore, there is very little validity to the *DSM–5* Section II categorical model. Problems with this model are well documented and manifold and will not be reviewed here. However, the major issues are (a) excessive between-category, between-cluster, and within-cluster comorbidity; (b) extreme clinical heterogeneity *within* diagnostic categories; (c) arbitrary diagnostic thresholds that do not adequately index severity; (d) inadequate coverage of the full range of personality pathology; (e) limited clinical utility; (f) no evidence for the existence of the discrete PD categories; (g) limited convergent validity; (h) temporal instability; and (i) inadequate diagnostic reliability (Cooper & Balsis, 2009; De Fruyt et al., 2013; Johansen, Karterud, Pedersen, Gude, & Falkum, 2004; Kotov et al., 2017; Lenzenweger, Lane, Loranger, & Kessler, 2007; Markon, Krueger, & Watson, 2005; Morey, Benson, Busch, & Skodol, 2015; Morey, Krueger, & Skodol, 2013; Quilty, Ayearst, Chmielewski, Pollock, & Bagby, 2013; Sheets & Craighead, 2007; Skodol et al., 2002; Trull, Scheiderer, & Tomko, 2012; Van den Broeck et al., 2014; Verheul & Widiger, 2004; Watson, Stasik, Ro, & Clark, 2013; Wright et al., 2012; Wright & Simms, 2014; Zimmerman, Rothschild, & Chelminski, 2005; Zimmermann et al., 2014).

For these reasons, an alternative, empirically derived diagnostic system for PD was proposed by the *DSM–5* Personality and Personality Disorders Work Group (P&PDWG), and its general criteria for PD are closely related to those proposed by the ICD–11 Working Group for the Revision of Classification of Personality Disorders (APA, 2013; Tyrer, Reed, & Crawford, 2015).^1^ Its general criteria for PD are based on the emerging body of research showing that PD is characterized by (a) impairment in self and interpersonal functioning and (b) the presence of one or more pathological personality traits (APA, 2013; Bender, Morey, & Skodol, 2011; Morey et al., 2015; Morey et al., 2011; Skodol, 2012, 2014). This diagnostic system thus recognizes that PDs consist of common features (i.e., self/interpersonal functioning impairments) and specific features unique to individual patients (i.e., pathological traits). Self functioning involves the domains of *identity* and *self-direction*, and interpersonal functioning involves the domains of *empathy* and *intimacy*. The domain of *identity* consists of (a) experiencing oneself as unique, with clear boundaries between self and others; (b) stability of self-esteem and accuracy of self-appraisal; and (c) a capacity for, and ability to regulate, a range of emotional experiences. The domain of *self-direction* consists of (a) pursuing coherent and meaningful short- and long-term goals, (b) the utilization of constructive and prosocial internal standards of behavior, and (c) the ability to self-reflect productively. The domain of *empathy* consists of (a) comprehension and appreciation of others’ experiences and motivations, (b) tolerance of differing perspectives, and (c) understanding the effects of one’s own behavior on others. Finally, the domain of *intimacy* consists of (a) depth and duration of connection with others, (b) a desire and capacity for closeness, and (c) mutuality of regard reflected in interpersonal behavior.

**Figure 1.**
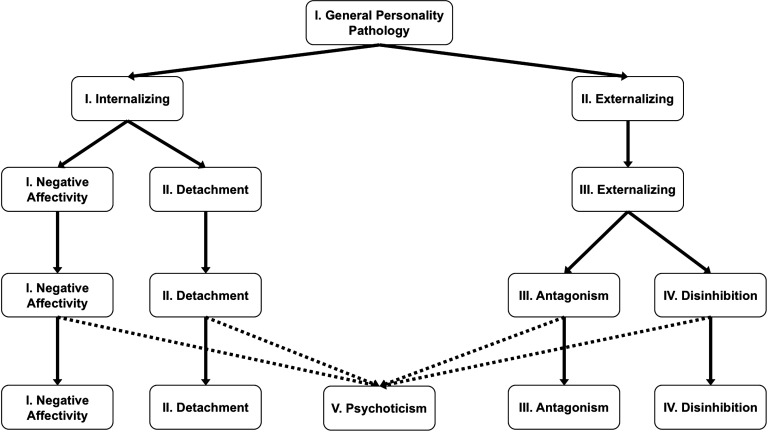
**Schematic representation of the hierarchical structure of personality pathology.** Two superordinate factors, called *Internalizing* and *Externalizing*, emerge from a general personality factor. Below this level there are three intermediate factors, for *Internalizing* splits into two lower-order factors labeled *Detachment* and *Negative Affectivity* whereas *Externalizing* maintains its structure. This three factor solution can then be decomposed into the five factors of *Negative Affectivity*, *Detachment*, *Antagonism*, *Disinhibition* and *Psychoticism*, each containing the lower-order trait facets that load on their respective trait domains. *Psychoticism* has complex links to the superordinate factors and this relationship is currently under investigation. Figure based on Wright et al. (2012).

The second feature of PD is the presence of pathological traits (e.g., emotional lability, depressivity, grandiosity, callousness, impulsivity). Factor analyses consistently reveal that pathological personality traits are organized into five dimensional factors reflecting the pathological ends of the five-factor model (FFM) of normal personality (De Fruyt et al., 2013; Markon et al., 2005; Morey, Krueger et al., 2013; Quilty et al., 2013; Sheets & Craighead, 2007; Van den Broeck et al., 2014; Watson et al., 2013; Wright & Simms, 2014, 2015; Wright et al., 2012; Zimmermann et al., 2014): (a) *Negative Affectivity* = *Neuroticism*, (b) *Detachment* = inverse of *Extraversion*, (c) *Antagonism* = inverse of *Agreeableness*, (d) *Disinhibition* = inverse of *Conscientiousness* and (e) *Psychoticism*, which reflects odd/eccentric/unusual behaviors and cognitions characteristic of schizotypal personality traits. The relationship between *Psychoticism* and *Openness* (which is not strongly linked to PD; Samuel & Widiger, 2008) is complex and currently under investigation. What is critical is that these factor analyses show that PD (a) is hierarchically organized (Figure 1), (b) exists on a *continuum* with the core dimensions of normal personality, and (c) has a structure that shares a strong resemblance to the factor structure of general psychopathology (Caspi et al., 2014; Keyes et al., 2013; Kotov et al., 2011, 2017; Krueger & Markon, 2006; Lahey et al., 2012; Markon, 2010; Wright et al., 2013).

Thus, a person can be said to have a PD when they have significant impairment in their identity, self-direction, capacity for empathy and/or intimacy, along with maladaptive personality trait(s) from the *Negative Affectivity*, *Detachment*, *Antagonism*, *Disinhibition* and/or *Psychoticism* dimensions of personality (APA, 2013). The unique profile of personality traits and impairment in self/interpersonal functioning is what differentiates different *styles* of PD from each other (e.g., borderline PD vs. psychopathic PD).

With this in mind, we can now approach an operational definition of psychopathy in this larger context of PD. Patients with psychopathy are typically described as lacking remorse, callous, grandiose, manipulative, superficially charming, impulsive, and prone to violent and antisocial behavior (Hare & Neumann, 2008). While psychopathy is one of the most extensively studied PDs, debates continue surrounding its nature and structure (Cooke, Hart, Logan, & Michie, 2012; Cooke & Michie, 2001; Hare & Neumann, 2008; Poythress & Hall, 2011; Sellbom, Cooke, & Hart, 2015; Skeem & Cooke, 2010). To make headway on an empirical understanding of the nature/structure of psychopathy, Cooke et al. (2012) developed a concept map of psychopathy based on an extensive literature review and consultations with experts in the field. The result of this process was the *Comprehensive Assessment of Psychopathic Personality* (CAPP; Cooke et al., 2012; Cooke et al., 2004), which identifies 33 personality traits that were translated into nontechnical language and rationally grouped into six domains (Table 1). These domains are the *Self*, *Emotional*, *Dominance*, *Attachment*, *Behavioral*, and *Cognitive* domains. The CAPP provides an adequate descriptive account of the psychopathy construct, as evidenced by professionals’ prototypicality ratings (Flórez et al., 2015; Hoff, Rypdal, Mykletun, & Cooke, 2012; Kreis & Cooke, 2011; Kreis, Cooke, Michie, Hoff, & Logan, 2012). Further evidence that the CAPP adequately covers the relevant traits comes from the large correlations (*r* = 0.66–0.73) between the CAPP domains and the *Psychopathy Checklist–Revised* (PCL–R; Sandvik et al., 2012), which is undoubtedly the most validated assessment of psychopathy (Hare, 2003; Hare & Neumann, 2008; Leistico et al., 2008; Patrick, 2006).

**Table 1.** Comprehensive Assessment of Psychopathic Personality (CAPP) traits and domains

**Domain**	**Traits**
Self	Self-centered
	Self-aggrandizing
	Sense of uniqueness
	Sense of entitlement
	Sense of invulnerability
	Self-justifying
	Unstable self-concept

Emotional	Lacks anxiety
	Lacks pleasure
	Lacks emotional depth
	Lacks emotional stability
	Lacks remorse

Dominance	Antagonistic
	Domineering
	Deceitful
	Manipulative
	Insincere
	Garrulous

Attachment	Detached
	Uncommitted
	Unempathic
	Uncaring

Behavioral	Lacks perseverance
	Unreliable
	Reckless
	Restless
	Disruptive
	Aggressive

Cognitive	Suspicious
	Lacks concentration
	Intolerant
	Inflexible
	Lacks planfulness

Despite ongoing controversies, three major findings have emerged about the nature/structure of psychopathy. First, whether assessed using clinician-rated or self-report tools, there is now convincing evidence that psychopathy is *not* a class or discrete category; rather, it is a dimensional construct (Edens, Marcus, Lilienfeld, & Poythress, 2006; Guay, Ruscio, Knight, & Hare, 2007; Hare & Neumann, 2008; Marcus, John, & Edens, 2004; Marcus, Lilienfeld, Edens, & Poythress, 2006; Walters et al., 2007). That is, “a psychopath” is technically a misnomer, insofar as it suggests someone who is qualitatively different from other people. Rather, psychopathy reflects a cluster of pathological personality traits that exist on a *continuum* with normal personality traits. Second, although there are differences depending on what measure is used, there is convergence about the structure of psychopathy. Factor analyses of the PCL–R suggest that psychopathy consists of a superordinate psychopathy factor comprising two higher-order correlated factors called Factor 1 (F1) and Factor 2 (F2), which further decompose into four lower-order correlated factors or facets (Hare & Neumann, 2008; Neumann, Hare, & Newman, 2007). F1 contains the *Interpersonal* (e.g., grandiosity, conning/manipulative) and *Affective* factors (e.g., lacks remorse, shallow affect), and F2 contains the *Lifestyle* (e.g., stimulation seeking, impulsivity) and *Antisocial* factors (e.g., poor behavioral controls, criminal versatility). The CAPP traits converge with the PCL-R in meaningful ways (Sandvik et al., 2012). Specifically, F1 is highly correlated (*r* > 0.60) with traits from the *Self*, *Dominance*, and *Attachment* domains, whereas the *Emotional* domain is highly correlated with the *Affective* sub-factor specifically. Traits from the *Behavioral* domain are highly correlated with F2, whereas the *Cognitive* domain is highly correlated with the *Lifestyle* sub-factor specifically. Self-report measures of psychopathy have been developed and also support this two-factor/four-factor model, notably the *Self-Report Psychopathy Scale III* (SRP–III; Williams, Paulhus, & Hare, 2007), the *Levinson Self-Report Psychopathy Scale* (LSRP; Levenson, Kiehl, & Fitzpatrick, 1995; Salekin, Chen, Sellbom, Lester, & MacDougall, 2014), and the *Antisocial Process Screening Device* (APSD; Frick & Hare, 2001; Vitacco, Rogers, & Neumann, 2003).

There are, however, points of disagreement about the structure of psychopathy worth describing briefly. Based on clinical formulations of psychopathy and confirmatory factor analyses, Cooke and Michie (2001) argued that the antisocial factor is not part of the core construct but rather is merely a correlate or consequence of a psychopathic personality. Using the PCL-R, they proposed a three-factor model with the antisocial items removed, leaving the remaining traits grouping into the factors *Arrogant and Deceitful Interpersonal Style*, *Deficient Affective Experience*, and *Impulsive and Irresponsible Behavioral Style* (Cooke & Michie, 2001). This three-factor model corresponds to the *Interpersonal*, *Affective*, and *Lifestyle* factors, respectively, of the original four-factor model. While Cooke and Michie (2001) emphasized their disagreement about the centrality of the *Antisocial* factor, their model is more alike than different. Two other prominent models of psychopathy are (a) the Triarchic model (Patrick, Fowles, & Krueger, 2009) and (2) the two-factor model of the *Psychopathic Personality Inventory* (PPI) and its revision, the PPI–R (Lilienfeld & Andrews, 1996; Lilienfeld & Widows, 2005). Based on a review of the clinical and empirical literature, the Triarchic model identifies *Disinhibition*, *Boldness*, and *Meanness* as the essential factors of the construct (Patrick et al., 2009). The PPI is composed of two higher-order factors called *Fearless Dominance* (PPI-FD) and *Self-Centered Impulsivity* (PPI–SCI; Benning, Patrick, Hicks, Blonigen, & Krueger, 2003; Lilienfeld & Widows, 2005).

The *Meanness* and *Disinhibition* factors of the Triarchic model capture traits associated with F1 and F2, respectively, of the PCL-R (J. Anderson, Sellbom, Wygant, Salekin, & Krueger, 2014; Patrick et al., 2009; Sellbom & Phillips, 2013; Stanley, Wygant, & Sellbom, 2013). Similarly, meta-analytic evidence shows that PPI–SCI is associated with F1 (*r* = 0.20–0.38), F2 traits (*r* = 0.41–0.57) and total PCL (*r* = 0.51) scores—however PPI–SCI is evidently more strongly linked to F2 traits (Marcus, Fulton, & Edens, 2013; Miller & Lynam, 2012). By contrast, measures of “boldness” (e.g., PPI-FD, Triarchic *Boldness*) are at best modestly correlated with measures of psychopathy (J. Anderson et al., 2014; Lynam & Miller, 2012; Miller & Lynam, 2012; Sellbom & Phillips, 2013; Stanley et al., 2013). Meta-analytic research shows that the PPI–FD is weakly correlated with F1 (*r* = 0.21–0.23), F2 (*r* = 0.07–0.15) and total PCL (*r* = 0.16) scores (Marcus et al., 2013; Miller & Lynam, 2012). Indeed, prior meta-analyses of boldness found little evidence that boldness is associated with known correlates of psychopathy (e.g., violent/antisocial behavior, substance use) or with functional impairment, for the associations with FFM traits show that people high in boldness can be described as emotionally stable, calm, and even-tempered (i.e., low *Negative Affectivity*), as well as sociable, warm, cheerful, and assertive (i.e., high *Extraversion*; Marcus et al., 2013; Miller & Lynam, 2012). There is even evidence that boldness is associated with *Openness* (*r* = 0.36; Patrick & Drislane, 2015), which has no relationship to psychopathy (*r* = −0.02; Decuyper, De Pauw, De Fruyt, De Bolle, & De Clercq, 2009). Thus, boldness can be said to reflect *emotionally stable extraversion* rather than psychopathy, and thus is not a core feature of psychopathy (Lynam & Miller, 2012; Miller & Lynam, 2012).

Taken together, there is general agreement in the field that psychopathy has a hierarchical factor structure consisting of a superordinate psychopathy factor and two higher-order correlated factors that are essentially identical to F1 and F2 of the PCL-R. Specifically, the first factor consists of traits captured by the *Interpersonal* and *Affective* factors of the PCL-R, the *Self*, *Dominance*, *Emotional*, and *Attachment* domains of the CAPP, and *Meanness* of the Triarchic model. The second factor consists of traits captured by the *Lifestyle* factor of the PCL-R, the *Behavioral* and *Cognitive* domains of the CAPP, *Disinhibition* of the Triarchic model, and the SCI factor of the PPI.

Most striking is that these two factors reflect maladaptive traits that correspond almost identically to the factors *Antagonism* and *Disinhibition*, respectively. This is unsurprising given that meta-analyses and expert ratings of the FFM consistently find that psychopathy principally reflects the inverse of *Agreeableness* (*r* = −0.55; i.e., *Antagonism*) and, to a lesser degree, the inverse of *Conscientiousness* (*r* = −0.34; i.e., *Disinhibition*; Decuyper et al., 2009; Lynam & Miller, 2015). It is also in keeping with factor analyses consistently revealing that a “psychopathy factor” is a basic dimension of abnormal personality (Kushner, Quilty, Tackett, & Bagby, 2011; Livesley, 2011; Markon et al., 2005; Morey, Krueger et al., 2013; Wright et al., 2012). The psychopathy factor is shown in Figure 1 under the label of *Antagonism*, within the superordinate *Externalizing* factor of personality pathology (APA, 2013). Alternative labels for the psychopathy factor in the literature are the *dyssocial domain* (ICD–11; Tyrer et al., 2015) and *Dissocial Behavior* (DAPP–BQ; Livesley & Jackson, 2009). Thus, the superordinate psychopathy factor can be reconceptualized as reflecting the general *Externalizing* factor of personality pathology (Figure 1)—a point already suggested by Hare and colleagues (Hare & Neumann, 2008; Neumann et al., 2007).

This dimensional perspective is important because it may resolve a long-standing debate about whether or not antisocial/criminal behavior and impulsivity are core components of psychopathy (Hare & Neumann, 2008, 2010; Poythress & Hall, 2011; Skeem & Cooke, 2010). The research above has suggested that, whereas the F1 traits of the PCL-R reflect the *Antagonism* dimension of personality pathology, the F2 traits reflect the *Disinhibition* dimension. Furthermore, the superordinate psychopathy factor reflects the general *Externalizing* dimension of personality pathology. Thus, F1 and F2 traits may be considered part of the same construct, insofar as psychopathy—broadly speaking—reflects the *Externalizing* dimension of personality pathology (Hare & Neumann, 2008; Neumann et al., 2007). This common superordinate factor explains why F1 and F2 scores are strongly positively correlated with each other (Hare & Neumann, 2008). The superordinate factor of *Externalizing* also accounts for the meta-analytic evidence that F1 and F2 scores are *both* moderately associated with general and violent (including sexual) offending and institutional misconduct (Leistico et al., 2008). This is unsurprising given the meta-analytic research showing that the inverse of *Agreeableness* (i.e., *Antagonism*) and the inverse of *Conscientiousness* (i.e., *Disinhibition*) are the strongest FFM personality predictors of violent/antisocial behavior (S. E. Jones, Miller, & Lynam, 2011; Miller & Lynam, 2001). On the other hand, given that psychopathy is more strongly correlated with *Antagonism* vs. *Disinhibition* (Decuyper et al., 2009; Lynam & Miller, 2015), strictly speaking, *Antagonism* (i.e., F1 traits) likely forms the core of the psychopathic personality, a point which has been suggested by others (Poythress & Hall, 2011; Skeem & Cooke, 2010). This is in keeping with factor analyses of the CAPP and prototypicality ratings of psychopathy—by mental health and correctional professionals—which show that traits associated with the *Self*, *Attachment*, and *Dominance* domains of the CAPP are the core personality traits of the disorder (Flórez et al., 2015; Hoff et al., 2012; Kreis & Cooke, 2011; Kreis et al., 2012; Sellbom et al., 2015; Sörman et al., 2014).

A working operational definition of psychopathy can therefore be proffered, based on the aforementioned evidence on the nature/structure of psychopathy and PDs more generally. A patient can be said to have a psychopathic personality when they have high levels of *Antagonism* traits, which may or may not co-occur with *Disinhibition* traits. These traits are specific expressions of a more general impairment in their self/interpersonal functioning, such that the patient’s *identity*, *self-direction*, capacity for *empathy* and/or *intimacy* is characterized by grandiosity, egocentricity, absent or few prosocial internal standards, limited self-reflection, difficulties understanding/appreciating other’s experiences, callousness, and/or limited mutuality in relationships. There is limited mutuality because the patient’s relationships are conceptualized largely in terms of meeting their self-regulatory and self-esteem needs, leading to manipulative, domineering, and/or uncommitted/detached relations with others.

Our article focuses on modeling *lacks remorse* and *self-aggrandizing* because these two traits are consistently ranked among the most prototypical traits of psychopathic PD in psychometric research (Cooke & Michie, 2001; Hare & Neumann, 2008; Sellbom et al., 2015) and in surveys of mental health and correctional professionals’ expert opinions about this PD (Flórez et al., 2015; Hoff et al., 2012; Kreis & Cooke, 2011; Kreis et al., 2012). Furthermore, these traits load on the *Antagonism*domain of personality pathology (APA, 2013; Kotov et al., 2017; Livesley, 2011; Wright et al., 2012), the core cluster of traits of the psychopathic personality (Poythress & Hall, 2011; Skeem & Cooke, 2010). *Lacks remorse* is a trait manifested by individuals described as unrepentant, unapologetic, or unashamed (e.g., denies having hurt others or minimizes the consequences for the victim, blames harmful behavior on others), whereas individuals with the trait *self-aggrandizing* are described as self-important, conceited, or condescending (e.g., regards self as being of higher status, dismissive toward those they consider beneath them; Cooke et al., 2004). Furthermore, as we will see, the pathogenesis of these traits has been the focus of significant theorizing within the psychotherapeutic literature. This is important because these psychotherapeutic models provide us with a rich and clinically relevant theoretical framework that can be formalized in terms of (active) inference and predictive coding. Our model, therefore, is not a complete account of psychopathy, for many traits still need to be formalized (Table 1). Rather, we believe that this model provides an initial proof of concept of the utility of the inference framework to understanding psychopathy. We illustrate this utility by showing that the two cardinal traits of *lacks remorse* and *self-aggrandizing* can be modeled in terms of abnormal Bayesian inference. It is our hope that other traits will yield to a similar formulation under this framework.

## PSYCHOTHERAPEUTIC MODELS OF PSYCHOPATHY

Psychotherapeutic models contribute to psychometric accounts of psychopathy by providing etiological frameworks to explain the pathogenesis of PDs. As we will demonstrate, there is a great deal of commonality between the cognitive and psychodynamic models of psychopathy, particularly with regard to their joint emphasis on (a) the importance of early adverse attachment experiences interacting with genetic vulnerabilities in developing a psychopathic patient’s core self-image as worthless and shameful and (b) how psychopathic traits can be understood as maladaptive “defense mechanisms” or “coping strategies” to this profoundly negative core self-image. These commonalities suggest that an integrated psychotherapeutic perspective is emerging. This psychotherapeutic account will be important, because it provides the theoretical constructs for formalizing *lacks remorse* and *self-aggrandizing* in terms of abnormal Bayesian inference.

### Psychodynamic Models of Psychopathy

In psychodynamic theory, patients with psychopathy are often described as having deep-seated self-related negative emotions, particularly feelings of worthlessness and shame. These feelings are rooted in their early experiences of being devalued, made to feel inadequate and unacceptable in the eyes of their attachment figures (Gacono, Meloy, & Berg, 1992; Kernberg, 1985; Kohut, 1966, 1977; Meloy & Shiva, 2007; Perry, Presniak, & Olson, 2013). During development, the first mental representations of the self and others are constructed from these early interactions with attachment figures, called *internal working models* (IWMs), which often operate largely unconsciously (Bowlby, 1969, 1973; Pietromonaco & Barrett, 2000). Psychopathic patients’ adverse attachment experiences lead to the formation of an IWM of the self characterized as worthless and shameful, which influences how these patients subsequently interpret information and regulate their self-esteem, emotions, and behavior (Lorenzini & Fonagy, 2013). Consistent with this, there is robust evidence that experiences precluding the formation of secure attachments, such as abuse, neglect, parental separation, and parental dysfunction, are risk factors for psychopathy and for violent/antisocial behavior (Campbell, Porter, & Santor, 2004; Cohen et al., 2014; Craparo, Schimmenti, & Caretti, 2013; Douglas, Hart, Webster, & Belfrage, 2013; Graham, Kimonis, Wasserman, & Kline, 2012; Hoeve et al., 2009; Johnson, Cohen, Brown, Smailes, & Bernstein, 1999; Kolla et al., 2013; Luntz & Widom, 1994; Marshall & Cooke, 1999; National Institute of Health and Clinical Excellence, 2010; Poythress, Skeem, & Lilienfeld, 2006; Roberts, Yang, Zhang, & Coid, 2008).

From a psychodynamic perspective, psychopathic personality traits reflect the operation of defense mechanisms that protect against the conscious experience of powerful self-related negative emotions arising from the IWM of the self. Specifically, individuals with psychopathy use denial, rationalization, and projection to disavow these negative experiences (Perry et al., 2013). Moreover, they construct a grandiose self-image to distort their self-appraisal and devalue and act aggressively toward others who threaten their grandiose veneer. Self-aggrandizement thus distorts the patient’s self-image to defend against their painful feelings of shame and worthlessness arising from their IWM of the self. Similarly, a lack of remorse defends against the conscious experience of these negative self-related emotions after breaching social norms (e.g., committing violent or antisocial acts) by denying having hurt others or minimizing the consequences of their actions. Lacking remorse thus serves a vital function for patients, because they are extremely sensitive to feeling worthless/shameful and will do anything to bypass or diminish these emotions. Such defensive functioning can take the form of hostility and violence, which can restore a patient’s self-esteem by making the perceived perpetrator of the shaming feel vulnerable and powerless (Logan & Johnstone, 2010). The extreme end of such reactive aggression is homicide. Patients’ low tolerance for these negative emotions is well known in the correctional/forensic rehabilitation literature (C. M. Jones, 2014; Maruna & Ramsden, 2004; Walker & Bright, 2009) and likely has its roots in the IWM of the self, which developed from the patients’ early adverse attachment experiences.

### Cognitive Models of Psychopathy

Although described using different terms, cognitive models of psychopathy have much in common with this psychodynamic formulation. The two major models are Davidson’s (2007) cognitive-behavioral therapy (CBT) model and the schema focused therapy (SFT) model (Bernstein, Arntz, & de Vos, 2007; Young, Klosko, & Weishaar, 2003). Davidson (2007) developed CBT for PDs, which formed the basis of the psychotherapeutic intervention of the Chromis programme for treatment of high-risk offenders in the United Kingdom with high levels of psychopathic traits (Tew & Atkinson, 2013). The CBT model integrates the traditional cognitive model of psychopathology with attachment theory, developmental psychology, and Beck and colleagues’ (A. T. Beck & Freeman, 1990; A. T. Beck, Freeman, & Davis, 2004) evolutionary perspective on PDs. The cognitive model understands psychopathology as emerging across three levels of belief: (a) *schemas* or “*core beliefs*,” (b) *intermediate beliefs*, and (c) *automatic thoughts* (A. T. Beck, 1964; A. T. Beck & Freeman, 1990; A. T. Beck et al., 2004; J. S. Beck, 2011). *Schemas* are the most basic structures that organize our experience and construct meaning out of our perceptions. Schemas play a fundamental and global role in information processing because they contain core beliefs about the self, others, and the world, which ultimately influence how patients think, feel, and behave moment by moment. For this reason, schemas are often not consciously articulated or accessible and thus operate outside conscious awareness. *Intermediate beliefs* are more consciously articulated and accessible rules, attitudes, and assumptions about oneself, others, and the world (e.g., “You can’t trust people”). Intermediate beliefs are thus a bridge between schemas and the final level of belief: automatic thoughts. *Automatic thoughts* are the most superficial level of cognition, because they are the reflexive thoughts that enter consciousness in response to situations that activate underlying schemas and intermediate beliefs. Automatic thoughts reflect the deeper layers of belief and directly influence how a patient feels and behaves. The CBT model of PD integrates this traditional cognitive model with an evolutionary perspective, because it is hypothesized that these schemas can also activate innate *behavioral strategies*, which are genetically determined behavioral patterns (e.g., aggression) that promoted survival and reproduction throughout most of human evolution (A. T. Beck & Freeman, 1990; A. T. Beck et al., 2004). Key to the CBT model of PDs is the idea that the interaction between a person’s genetic predispositions (e.g., temperament, neuropsychological functioning) and childhood environment shapes the development of the person’s schemas and behavioral strategies. If a primary caregiver is emotionally unavailable to their child, insensitive or unresponsive to the child’s emotional needs, or has a chaotic or harsh parenting style, the child will likely develop an insecure attachment style as a consequence of developing dysfunctional IWMs (i.e., core beliefs) about themselves and others (Bowlby, 1973; Davidson, 2007; Pietromonaco & Barrett, 2000). Throughout development, patients with PD learn a variety of strategies to cope with their core beliefs of low self-worth, vulnerability, and being unloved. Furthermore, an adverse environment can amplify or inhibit the innate behavioral strategies in patients with PDs, such that they can become overdeveloped or underdeveloped and thus maladaptive in modern social environments.

From the CBT model’s perspective, the early adverse attachment experiences psychopathic patients encounter interact with their genetic vulnerabilities, resulting in the formation and reinforcement of dysfunctional schemas, such that they have a core belief about themselves as being unworthy, powerless, and unloved (Davidson, 2007). Additionally, consistent with the psychodynamic model, compensatory intermediate beliefs develop as a way to cope with these deep feelings of worthlessness and inadequacy. For instance, patients typically form egocentric and self-aggrandizing beliefs that they are strong, can do whatever they want, and are entitled to exploit others and thus show a lack of remorse for their antisocial behavior (A. T. Beck & Freeman, 1990; A. T. Beck et al., 2004). Compensatory coping strategies can also involve innate behavioral strategies. For example, patients learn to use hostility and violence to avoid being perceived by others as weak, which triggers intolerable anxiety and feelings of vulnerability, resulting in the overdevelopment of aggression (Davidson, 2007).

SFT has similarly been adapted for use in forensic psychiatric hospitals in the Netherlands, called *Terbeschikkingstelling* (TBS) clinics, for the purpose of treating forensic patients with severe PDs, particularly patients with psychopathy (Bernstein et al., 2007). While not exclusively a cognitive model—because it combines cognitive, behavioral, psychodynamic, and existential/humanistic approaches—the SFT model has prominent features of the traditional cognitive model of psychopathology. There are three major components to the SFT model of PDs: Early Maladaptive Schemas (EMSs), coping strategies, and Schema Modes (Young et al., 2003). EMSs are the core pathology of patients with PDs, and they develop out of the interaction between genetic vulnerabilities (e.g., temperament) and unmet emotional needs the patient experienced early in life.

Like IWMs and core beliefs, EMSs are highly stable structures that organize a person’s core self-identity and mental representations of others around specific themes and are elaborated throughout life. The SFT model identifies 18 EMSs, which are grouped into five domains, reflecting failures to meet the five universal emotional needs (Table 2). Patients experience powerful self-related negative emotions (e.g., shame, vulnerability, self-hatred) when an EMS is activated. Patients attempt to eliminate, or at least diminish, these negative emotions using three coping strategies: Schema Maintenance, Schema Avoidance, and Schema Compensation. In Schema Maintenance, the patient reinforces their schema by discounting information that would disconfirm their EMS through cognitive distortions or self-defeating behavior. In Schema Avoidance, the patient attempts to suppress thoughts or feelings or behaviorally avoid situations associated with their EMS. In Schema Compensation, the patient overcompensates for the negative emotions by acting or generating emotions in the polar opposite direction of the content of their EMS.

**Table 2.** Early maladaptive schemas and schema domains

**Basic emotional need**	**Schema domain**	**Early maladaptive schemas**
1. Secure attachments to others	Disconnection and rejection	1. Abandonment/instability
		2. Mistrust/abuse
		3. Emotional deprivation
		4. Defectiveness/shame
		5. Social isolation/alienation
2. Autonomy, competence and sense of identity	Impaired autonomy and performance	6. Dependence/incompetence
		7. Vulnerability to harm or illness
		8. Enmeshment/undeveloped self
		9. Failure
3. Realistic limits and self-control	Impaired limits	10. Entitlement/grandiosity
		11. Insufficient self-control/self-discipline
4. Freedom to express valid needs and emotions	Other-directedness	12. Subjugation
		13. Self-sacrifice
		14. Approval-seeking/recognition seeking
5. Spontaneity and play	Over-vigilance and inhibition	15. Negativity/pessimism
		16. Emotional inhibition
		17. Unrelenting standards/hypercriticalness
		18. Punitiveness

Schema Modes are defined as those schemas and coping strategies that are currently dominating the moment to moment thoughts, feelings and behavior of a person. Young et al. (2003) originally identified 11 Schema Modes; however, as a consequence of their therapeutic work with personality disordered forensic patients, Bernstein et al. (2007) expanded the list to include four new Schema Modes (Table 3). Patients with psychopathy are characterized by prominent use of four Schema Modes that involve compensatory coping responses to their EMSs, particularly those EMSs with themes of disconnection and rejection (Bernstein et al., 2007). Specifically, they predominantly use the *Self-aggrandizer mode*, *Bully and attack mode*, *Conning and manipulative mode*, and *Predator mode* as a way to overcompensate for feelings of shame, loneliness and vulnerability. The *Self-aggrandizer mode* allows the patient to distort their conscious thoughts and feelings about their self in order to “defend against” or “cope with” their deep self-related negative emotions arising from their underlying EMSs (i.e., IWM). Similarly, the *Predator mode* allows the patient to overcome deep feelings of shame and worthlessness by becoming a predator who can eliminate threats to their self-esteem without remorse and also command respect from others through fear (Bernstein et al., 2007).

**Table 3.** Schema modes

**Modes**	**Schemas**
**Child modes:** Involve feeling, thinking, and acting in a “childlike” manner	1. Vulnerable child (abandoned, abused, or humiliated child)
	2. Angry child
	3. Impulsive, undisciplined child
	4. Lonely child

**Dysfunctional coping modes:** Involve attempts to protect the self from pain through maladaptive forms of coping	5. Detached protector
	6. Detached self-soother/self-stimulator
	7. Compliant surrenderer
	8. Angry protector^*a*^

**Maladaptive parent modes:** Involve internalized dysfunctional parent “voices”	9. Punitive, critical parent
	10. Demanding parent

**Over-compensatory modes:** Involve extreme attempts to compensate for feelings of shame, loneliness, or vulnerability	11. Self-aggrandizer mode
	12. Bully and attack mode
	13. Conning and manipulative mode^*a*^
	14. Predator mode^*a*^
	15. Over-controller mode (paranoid and obsessive-compulsive types)^*a*^

a New Schema Mode added by Bernstein et al. (2007).

### Toward an Integrated Psychotherapeutic Model of Psychopathy

This convergence of psychodynamic, CBT, and SFT models of psychopathic traits—specifically *lacks remorse* and *self-aggrandizing*—is extremely striking. This is because it suggests that the psychotherapeutic field is moving toward an integrated model of these traits. This integrated model centers on two hypotheses:

1.  Early adverse attachment experiences interact with genetic vulnerabilities to shape the development of a psychopathic patient’s core self-image as worthless and shameful. This core self-image is the first self-identity the patient develops during childhood and adolescence, and it continues to be elaborated upon throughout life. Furthermore, this core self-image is often not consciously articulated or accessible and thus operates outside conscious awareness. Various models call this core self-image the IWM of the self, self-schema, core belief, or EMS.
2.  The traits *lacks remorse* and *self-aggrandizing* are maladaptive defense mechanisms that allow the patient with psychopathy to eliminate or at least diminish (i.e., cope with) the influence of their negative core self-image on their conscious experience. These defense mechanisms are reflected in more consciously articulated and accessible beliefs about the self. Various models call these more conscious beliefs intermediate beliefs or Schema Modes.

This integrated psychotherapeutic model of *lacks remorse* and *self-aggrandizing* will be the basis of the Bayesian formulation described in the section “A Bayesian Account of Psychopathy.” However, before outlining the model, it is necessary to first consider the broader theoretical context for understanding the predictive coding framework (Corlett & Fletcher, 2014; Friston, 2010; Friston & Kiebel, 2009; Friston, Stephan et al., 2014).

## PREDICTIVE CODING, ACTIVE INFERENCE, AND THE BAYESIAN BRAIN

Predictive coding can be regarded as a corollary of the free-energy principle. The free-energy principle starts with the truism that biological systems are a unique class of self-organizing systems that exhibit a generalized *homeostasis*; that is, they resist the natural tendency to disorder by maintaining their physiological and sensory states in constantly changing internal and external environments. This self-sustaining characteristic means that an organism’s states must have low *entropy* if it is to remain viable and adaptive in its environments. Entropy is the average “surprise” or uncertainty, which, from an organism’s perspective, is unexpected (i.e., unpredicted) states. *Surprise* thus depends on the predictions of the organism, for what is surprising for one organism (e.g., being a fish out of water) may not be for another (Friston, 2010). This is important because it means that an organism’s evolutionary imperative of maintaining homeostasis (i.e., *survival*) can be accomplished if it minimizes its long-term average surprise, because failing to minimize surprise will necessarily lead to an increase in entropy (i.e., disorder) in the system.

In this context, surprise can be minimized by minimizing free-energy or—put simply—minimizing prediction error. Organisms minimize prediction error either by changing their predictions about how inputs are caused so that predictions match inputs, or through action that changes inputs so that they are consistent with predictions. This is known as *active inference*. The upshot is that by minimizing the discrepancy between an organism’s predictions and the actual inputs it receives, an organism can minimize its long-term average surprise (i.e., uncertainty or entropy) and thus increase the probability that it will survive (Friston, 2009, 2010; Friston & Kiebel, 2009; Friston, Kilner, & Harrison, 2006).

This formalism might sound a bit mathematical and abstract—and difficult to connect to how we function as sentient agents with beliefs. However, one key insight connects the imperatives for survival to beliefs and inference. This insight rests upon the fact that surprise is mathematically the same thing as (negative log) Bayesian model evidence: As surprise is resolved, Bayesian model evidence is increased. This means that every living organism behaves as if it is a little statistician, analyzing its sensory data in exactly the same way that scientists evaluate the evidence for their hypotheses about how experimental data were caused. In fact, free-energy is used routinely in data analysis and Bayesian model comparison to find the best model or explanation for observed data. In short, the free-energy principle and its corollary—the Bayesian brain hypothesis—offers a formal framework within which to understand action and perception in terms of a subject’s explanations or probabilistic beliefs about how the world generates sensations. In this view, minimizing surprise is, literally, the search for evidence for one’s own existence, under *generative models* of our self.

Predictive coding can be viewed as an instantiation of this “self-evidencing” process (Hohwy, 2016), and much neurophysiological and neuroanatomical evidence suggests that the brain implements this evidence-gathering, surprise-reducing, uncertainty-resolving behavior (Bastos et al., 2012; Clark, 2013; Friston, 2005, 2009, 2010; Huang & Rao, 2011). One major neuroanatomical fact about the brain—crucial to predictive coding—is its hierarchical organization (Bastos et al., 2012; Friston, 2005). In the predictive coding framework, *expectation units* (associated with deep pyramidal cells) at each level in the processing hierarchy predict neural representations of expectations at lower levels of the hierarchy (with basic sensory inputs represented at the lowest levels). These top-down predictions are received by lower-level *prediction error units* (associated with superficial pyramidal cells), which compare predictions with the expectations at that level. When there is a mismatch, a prediction error signal is generated—which ascends the hierarchy to revise the higher-level representations in order to provide better predictions. These then explain away (minimize) prediction error in the level below, thereby resolving prediction error throughout the hierarchy and reducing surprise.

In summary, descending connections between levels convey top-down predictions, whereas ascending connections convey prediction errors. Prediction errors are minimized at each level of the processing hierarchy, making the network more accurate in terms of explaining the inputs it receives from the environment. For example, if you change your facial expression, my descending predictions of the visual input (in terms of contours and shading, say, around your eyes) may no longer be accurate—eliciting a visual prediction error. This prediction error will ascend the visual hierarchy to revise high-level expectations about the causes of visual input, for example, you are “smiling.” This updated expectation or hypothesis (i.e., you are smiling) then provides better predictions of visual input, ensuring prediction errors are minimized throughout the hierarchy (Strathearn, Li, Fonagy, & Montague, 2008).

How does predictive coding instantiate a form of Bayesian inference? In Bayesian probability, the *posterior* probability or belief after observing data is evaluated by combining *prior beliefs* (i.e., beliefs prior to observing the data) with the *likelihood* of the observed data. The definition of *belief* in this context is not the traditional one (i.e., a consciously held proposition); rather, beliefs are probability distributions (which may or may not be conscious) over some unknown state or attribute of the world (e.g., whether you are smiling or not). Bayesian beliefs thus function like hypotheses. They are generally characterized by their *expectation* or mean, describing the most likely value, and *precision* (or inverse variance), describing the expected confidence (or inverse uncertainty) associated with the belief. In predictive coding, prior beliefs are encoded by neural activity conveying top-down predictions from deep pyramidal cells at higher levels, whereas the likelihood is conveyed by bottom-up prediction error signals from superficial pyramidal cells from the level below. Posterior expectations are encoded at each level in the hierarchy and reflect the brain’s perception at that level of (hierarchical) abstraction.

In a hierarchical setting, this means that posterior expectations at one level function as prior beliefs for the level below, and prediction error signals at one level serve as inputs for the level above. When a conflict exists between inputs and prior beliefs, precision plays a vital role in how the brain resolves the discrepancy. This is because precision determines the relative weights they are afforded, when the posterior expectation at a given level is evaluated. If the prior belief is relatively precise—compared to ascending input—the posterior expectation will be closer to the mean of the prior. However, if the ascending input is relatively precise compared to the prior, it will dominate the posterior expectation. In other words, the relative precision of top-down prior beliefs and bottom-up inputs can dramatically influence the kinds of inferences we make. As we will see, abnormalities in the encoding of precision will be important for understanding the pathogenic mechanisms underlying *lacks remorse* and *self-aggrandizing*. The available neurobiological evidence suggests that precision is encoded by the synaptic gain of superficial pyramidal cells encoding prediction errors, which are controlled by neuromodulatory systems (e.g., dopaminergic, cholinergic) and/or synchronized neural activity (Feldman & Friston, 2010).

**Figure 2.**
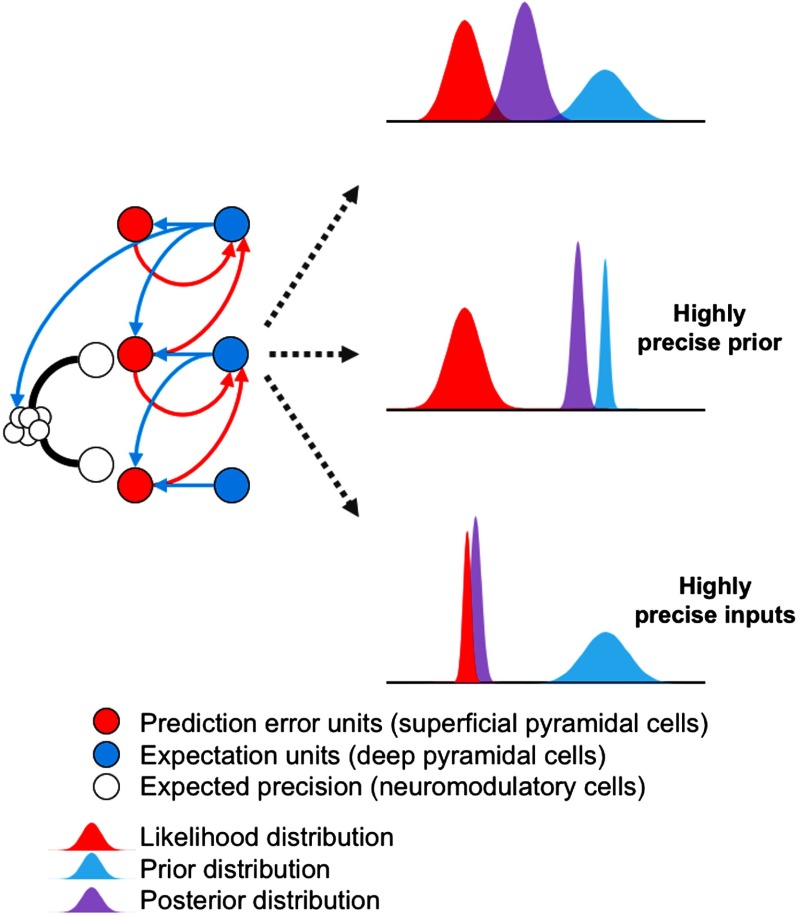
**Schematic of a predictive coding network and the role of precision in Bayesian inference.** See main text for details.

A schematic of predictive coding can help us understand these processes more concretely (Figure 2). The left panel displays how superficial pyramidal cells (red circles) encoding prediction errors are reciprocally connected at each level and between levels to deep pyramidal cells (blue circles), which encode top-down predictions for the level below and posterior expectations at each level. The expected precision is mediated by neuromodulatory cells whose projections to superficial pyramidal cells modulate their responsiveness or gain. The neurophysiological and neuroanatomical evidence suggests that these neuromodulatory pathways are under top-down influences (Baluch & Itti, 2011; Ferenczi et al., 2016; Friston, 2009, 2010; A. J. Yu & Dayan, 2005), which is indicated schematically by the descending connection from the highest level to the neuromodulatory cells. The right panel displays the probability distributions of the prior (blue), likelihood (red), and posterior (purple = blue + red) distributions at the intermediate level in the hierarchical network. The prior distribution is supplied by descending connections conveying the top-down predictions from the level above, whereas the likelihood distribution is supplied by the ascending connections conveying the prediction error signal from the level below. The top graph illustrates a situation where the ascending input—to an intermediate level—conflicts with top-down predictions, such that the brain’s perception (i.e., posterior expectation) of the input at that level is an approximately equal compromise between the two distributions. However, when neuromodulation increases prior precision—relative to sensory evidence—the posterior distribution shifts toward the prior and away from the likelihood. In other words, in this Bayesian synthesis, sensory evidence is effectively ignored by emphasizing prior beliefs. Conversely, when neuromodulators substantially increase the precision of the likelihood relative to the prior, the incoming data overpower top-down predictions, shifting the brain’s perception toward the input.

One can see why the process of optimizing expected precision has been associated with selective attention, because precision helps the brain to emphasize particular streams or aspects of sensory inputs, while selectively attenuating sensory precision to ignore evidence against prior beliefs. It is this particular aspect of Bayesian inference that appears to be the most likely candidate for explaining the false inferences and aberrant beliefs in psychopathology (Adams et al., 2013; Friston, Stephan et al., 2014). In other words, we do not necessarily have to have bad models of the world to entertain false beliefs. Rather, false inference can arise from a failure to properly balance the precision of prior expectations in relation to sensory evidence—or, more simply, an inability to attend to or ignore evidence that contradicts our prior expectations. The predictive coding framework has a special role here in connecting false inference to the neurophysiological processes that are implicated in augmenting or attenuating precision at different levels of the cortical hierarchy. Crucially, these processes necessarily involve synaptic neuromodulation and a pathophysiology of synaptic gain control or excitability of the sort seen in psychiatric conditions. Understanding false beliefs (e.g., delusions and hallucinations) in terms of abnormal neuromodulation underpins many recent treatments of psychiatric conditions, ranging from schizophrenia to functional (i.e., “hysterical”) symptoms (Adams et al., 2013; Corlett & Fletcher, 2014; Edwards et al., 2012; Friston, Stephan et al., 2014; Montague et al., 2012; Prosser et al., 2016). In what follows, we apply this line of argument to perhaps the most important prior beliefs we call upon, namely, beliefs about our self.

## A BAYESIAN ACCOUNT OF PSYCHOPATHY

**Figure 3.**
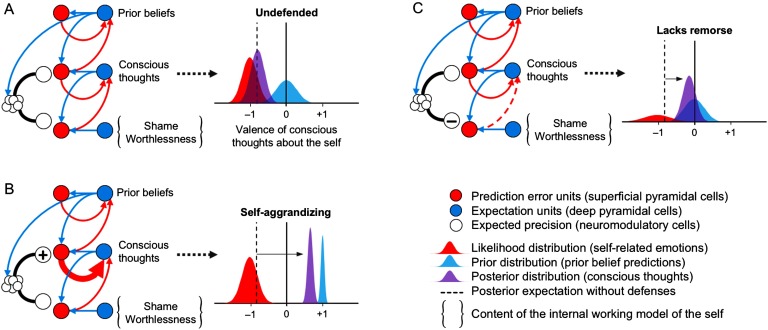
**Bayesian model of *lacks remorse* and *self-aggrandizing*.** See main text for details.

Predictive coding provides us with the conceptual resources to formalize in terms of Bayesian inference the integrated psychotherapeutic model of *lacks remorse* and *self-aggrandizing* outlined in the section “Psychotherapeutic Models of Psychopathy.” Specifically, these two psychopathic traits can be described under a hierarchical model of an embodied and prosocial self (Figure 3A). In this model, the valence (positive vs. negative) of conscious thoughts about the self is represented as an empirical prior^2^ or posterior belief, which integrates top-down predictions regarding self-appraisal with bottom-up affective signals from the *IWM of the self* (i.e., self-schema) from the level below. Therefore, much like the cognitive theory described in the section “Psychotherapeutic Models of Psychopathy” (A. T. Beck, 1964; A. T. Beck & Freeman, 1990; A. T. Beck et al., 2004; J. S. Beck, 2011), the *prior beliefs about the self* structure *automatic conscious thoughts* about the self in a top-down manner according to their predictions (i.e., the *content* of the beliefs). For this reason, these high-level prior beliefs are a formalization of the cognitive model’s concept of *intermediate beliefs*, which, recall, are more consciously articulated and accessible beliefs about oneself that shape conscious thoughts. Similarly, the *IWM of the self* supplies evidence about the self from lower levels of the hierarchy. However, these more basic self-representations are less consciously articulated and accessible and thus operate largely outside the person’s awareness (i.e., subpersonal beliefs). This formulation resonates closely with Bayesian approaches to self-representations (Moutoussis, Fearon, et al., 2014). Finally, neuromodulatory cells—which encode the precision of the top-down priors and bottom-up affective signals on conscious thoughts—are regulated in a top-down manner by descending connections in accord with high-level prior beliefs. Therefore we hypothesize that the high-level prior beliefs about the self are the source of the control signal for modulating the precision of bottom-up vs. top-down information about the self on conscious thoughts (i.e., posterior beliefs), which is indicated schematically in Figure 3 by the descending connection from the highest level (i.e., the level of the prior beliefs about the self) to the neuromodulatory cells. Clearly Figure 3 is a schematic representation, given that the computational and neurobiological details are likely much more complicated. Each one of these functionally distinct levels undoubtedly encompasses multiple networks of brain regions and neuromodulatory systems working in concert with one another. For the present purposes, however, it is sufficient to note that, even under these simplifying assumptions, this sort of hierarchical inference can illustrate how an integrated psychotherapeutic account of *lacks remorse* and *self-aggrandizing* may be formulated in a (neuronally plausible) computational architecture—as we elaborate next.

When undefended (Figure 3A), psychopathic patients’ conscious thoughts are overpowered by shame and worthlessness arising from their IWM of the self, resulting in automatic conscious thoughts having posterior expectations (purple distribution) that are shifted toward negative expectations about the self (red distribution) and away from their prior beliefs regarding self-appraisal (blue distribution).

When *self-aggrandizing* (Figure 3B), psychopathic patients defend against the conscious experience (posterior expectations) of shame and worthlessness arising from their IWM of the self via two mechanisms: top-down predictions from prior beliefs about the self are (a) abnormally elevated (prior distribution shifted rightwards) and (b) afforded too much precision (plus sign and thick red arrow). This results in automatic conscious thoughts having inflated positive posterior expectations about the self, despite the underlying presence of feelings of shame and worthlessness. Such descending neuromodulation by *self-aggrandizing* in effect ignores prediction errors that would otherwise provide contrary evidence against their prior grandiose beliefs about the self (e.g., feelings of shame/worthlessness). The neuromodulatory mechanism in this instance rests on augmenting the precision of these prior grandiose beliefs. In this way, the high-level prior beliefs about the self can be described as *compensatory* beliefs (or compensatory “intermediate beliefs,” in the cognitive model’s sense of the term) against a more basic, negative core self-schema (Davidson, 2007) by virtue of how they alter the posterior beliefs about the self at the level of conscious thoughts.

When showing a *lack of remorse* (Figure 3C), the patient defends against the conscious experience of shame and worthlessness (e.g., after breaching social norms) by decreasing the precision of the affective input on conscious thoughts (minus sign and dotted red arrow) via neuromodulatory systems controlling the synaptic gain on the affective signals from the IWM of the self. This results in automatic conscious thoughts having largely neutral (i.e., centered on zero valence) posterior expectations about the self despite socially inappropriate behavior. In this setting, the same defensive inference is in play (i.e., ignoring evidence that is contrary to prior beliefs about self)—however, here the mechanism involves an attenuation of the precision of ascending prediction error. To illustrate the subtle but malignant effect precision can have on inferences about the self and relations with others, we now turn to a simulation of psychopathy using dyadic interactions in simple game.

### Quantitative Simulations

In this section, we present some quantitative simulations of psychopathy to substantiate the hypotheses of the previous section; namely, that minimal impairments to the encoding of precision or uncertainty are sufficient to explain psychopathic traits and abnormal inferences about self-worth. We have until this point used predictive coding to illustrate hierarchical inference in the brain. However, we will use an equivalent—free-energy minimizing—active inference scheme, formulated in terms of discrete states. This is because discrete states are more apt for modeling the sorts of games and behaviors associated with trust and reputation formation (e.g., in behavioral economics), which can be used to simulate some of the claims we have made about psychopathic traits. Although the formalism of these schemes differs from predictive coding, the basic elements are conserved—namely, belief propagation using predictions and prediction errors to update expectations about the latent or hidden states of the world causing observable outcomes (Friston, FitzGerald, Rigoli, Schwartenbeck, & Pezzulo, 2017; Friston, Parr, & de Vries, 2017).

**Figure 4.**
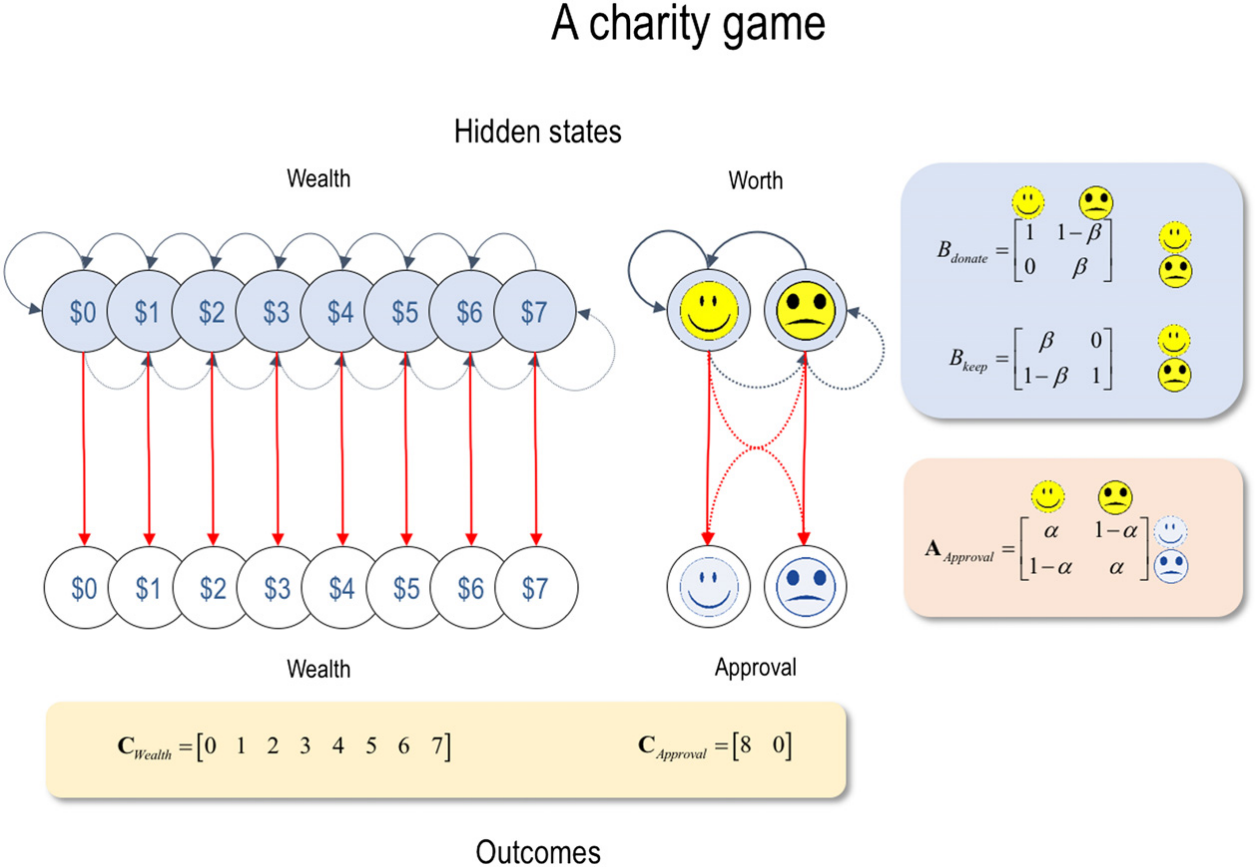
**Schematic overview of the generative model used to simulate a simple reputation game.** This model is shown in graphical form, where the circles correspond to different states (and outcomes) that a subject can be in (and observe). The red arrows correspond to the mapping between latent or hidden states and their observable consequences. This is the likelihood matrix (A) shown on the right. Transitions among these states are encoded by blue arrows (solid for donate and broken for keep). The corresponding probability transition (B) matrices are shown on the right. Finally, the preferences for outcomes are encoded in a (C) matrix. These preferences are specified in terms of log prior probabilities. In this model, *α* and *β* can be regarded as encoding the precision of sensory evidence and prior beliefs, respectively.

To illustrate the key role of precision at different levels of a hierarchical generative model, we used a Markov decision process (Friston, FitzGerald et al., 2017). Active inference under these models has been described in many previous applications, ranging from applications to choice behavior through to epistemic foraging and visual searches (Mirza, Adams, Mathys, & Friston, 2016; Parr & Friston, 2017; Schwartenbeck, FitzGerald, Mathys, Dolan, & Friston, 2014). Here, we use exactly the same formalism and (free-energy minimizing) update scheme to simulate a simple reputation game (Figure 4). In brief, the generative model underneath these sorts of simulations comprises hidden or latent states of the world and the outcomes that they generate. The mapping between states (i.e., causes) and outcomes (i.e., consequences) is described by a likelihood (A) matrix, whose precision we will associate with precision at the lower (e.g., sensory or perceptual) level of processing hierarchies. Transitions among hidden states depend upon policies or choices and are generally encoded in (choice dependent) probability transition (B) matrices. We will associate the precision of these matrices with prior precision—namely, the confidence placed in prior beliefs about state transitions under different choices. Finally, prior preferences over outcomes are encoded in a (C) matrix, in the form of log prior probabilities. These can be thought of as the value of different outcomes.

The game we modeled involved deciding whether to keep a sum of money or donate it to charity, depending upon how rich one is. The minimum requirements for this sort of game include hidden states that encode how nice or charitable the subject is, and how rich they believe themselves to be. Given these two hidden states, one can generate plausible outcomes. In this instance a (prosocial) feedback of *approval* or *disapproval*. In addition, we included the actual wealth of the subject as an observable feedback. In brief, our generative model contained two sorts of hidden states. The first was monetary *wealth* (with eight levels, ranging from *broke* to *wealthy*). The second hidden state was *self-worth* (with two levels: *charitable* versus *mean*). The observable outcomes had the same form: with one visual cue reporting the level of monetary *wealth* and another reporting *approval* or *disapproval* of the choice to donate or keep an offer on each trial. The likelihood (A) matrix was an identity mapping between the levels of the *wealth* factor. However, the mapping between the hidden state of *self-worth* and the *approval* cue could be precise or imprecise, depending upon the subject’s predisposition. In other words, the likelihood mapping could be deterministic, such that approval was always generated by a charitable state of being or it could be imprecise, such that there was a 50-50 chance of approval or disapproval irrespective of one’s *self-worth*. The prior probability transition matrices (B) contained the structure and dynamics of the game. These are specified separately (technically, conditioned upon) the two choices (*donate* or *keep*). For transitions among levels of wealth, every time the offer was donated, the level of wealth fell to the level below (or stayed at the lowest level). Conversely, if the choice was *keep*, the level of wealth increased (unless at the most wealthy state). In addition, we modeled an attrition of wealth, with a constant decay from higher levels to lower levels (with 10% probability of loss at each trial). Heuristically, this means the subjects were spending their wealth at a constant rate and could decide to accumulate more wealth by keeping the offer or, if they were sufficiently wealthy, increase the probability of an approval rating by donating.

The probability transition matrix among *self-worth* states controlled the rate at which *self-worth* changed from a *charitable* to a *mean* level (and *vice versa*), depending upon the actions selected. The parameterization of this matrix allowed us to modulate how behavior changed self-worth: here, donating ensured that a *charitable* self-worth was maintained, with a small probability of moving from a *mean* to a *charitable* state, and *vice versa* for keeping the offer. Finally, prior preferences were encoded in the (C) matrix for both outcome modalities. These comprised a log linear increase in prior preferences for being rich and a preference for approval over disapproval, both of which were fairly precise (with log prior differences in the range of four). The structure of this generative model and numerical examples of the likelihood (A), prior transition (B) and prior preference (C) matrices are provided in Figure 4.

Equipped with this model, we can now simulate choice behavior, starting from any prior expectations about self-worth and examine the long-term or equilibrium behavior. Here, we characterized behavior and underlying beliefs in terms of the average wealth retained by synthetic subjects—and the posterior expectation that they were *nice*. These simulations entailed 16 successive choices starting from a prior belief that they were charitable (but broke). By repeating the simulations under different levels of the likelihood precision (encoded by *α* Figure 4) and different precisions of the prior transition probabilities over self-worth (encoded by *β*), we could examine the effects of likelihood and prior precision on behavior and concomitant self-worth. By decreasing the precision of the likelihood (i.e., the confidence that approval ratings were a veridical reflection of one’s self-worth), we hoped to simulate *lacks remorse*, with an increasing tendency to keep offers. By increasing the precision of prior beliefs, we then hoped to simulate *self-aggrandizing*, in terms of *mean* behavior in the face of preserved self-worth.

**Figure 5.**
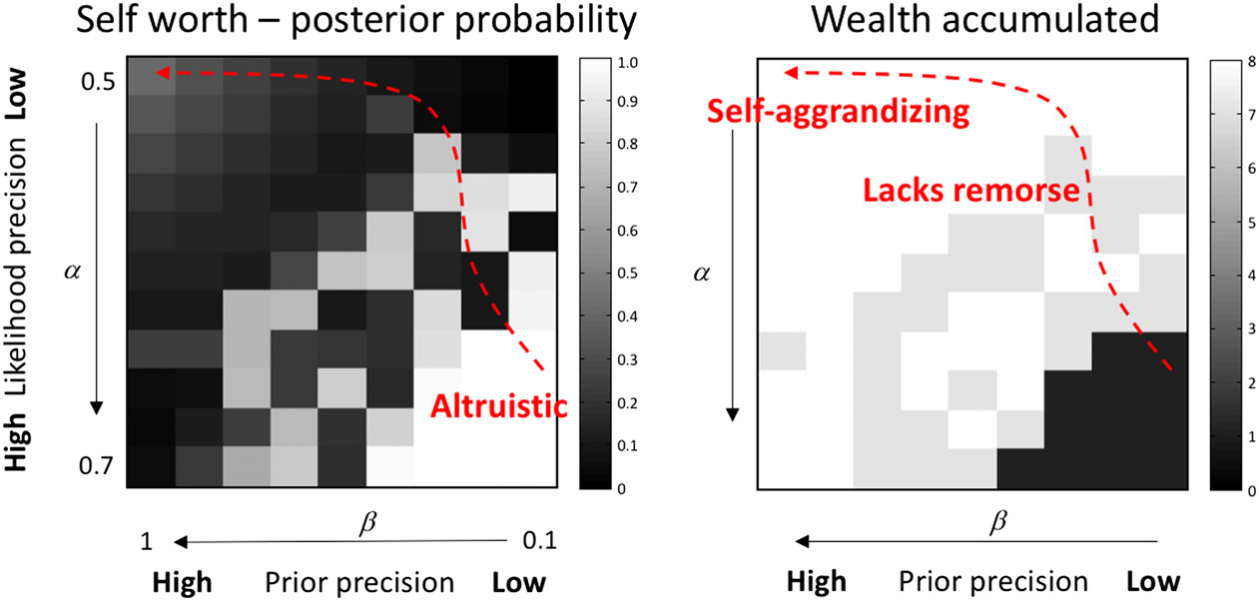
**This figure shows the effects of changing the likelihood precision (*α*) and transition precision (*β*) during a simulation of 16 choices starting from a prior expectation about being moneyless but nice.** This has been performed for synthetic subjects with different combinations of *α* and *β*. These effects are shown in terms of the posterior expectation about being charitable (left image) and the overall amount of wealth retained (right image). Although self-worth is generally lower for parameters that lead to greater wealth accumulation, the synthetic agents with very low *α* and high *β* (upper left corner of each plot) appear to have a higher posterior expectation of self-worth despite uncharitable behaviors. The developmental trajectory of a hypothetical patient with psychopathic traits is shown in red. Starting in the (altruistic) lower right corner, *α* initially decreases, uncoupling external sources of approval from beliefs about self. This trajectory leads to behaviors that yield greater monetary profit, but a lower self-worth. By subsequently increasing *β*, the influence of decisions on self-worth is diminished, uncoupling decisions from beliefs and resulting in uncharitable behavior that does not unduly compromise self-worth.

Figure 5 shows the results of these simulations as heat maps for self-worth (left panel) and wealth (right panel). As we would expect, synthetic subjects who accumulated the most wealth, by giving relatively few donations, had a lower posterior expectation that they were charitable. In other words, regions in the parameter space of likelihood and prior precision with high self-worth (white areas on the left) were associated with low wealth (dark areas on the right). Reducing *α*, or the degree to which self-worth reliably solicits approval, quickly reduces donations leading to an accumulation of wealth (under higher levels of prior precision). The interpretation of *β* is subtler.^3^ A very high value results in an identity matrix, de-coupling beliefs about transitions from choices. This corresponds to a high prior precision. Conversely, low values of *β* give rise to transitions that depend sensitively on actions. This corresponds to reduced prior precision. When we increase *β* (i.e., increase prior precision), the synthetic subject again accumulates more wealth, as the consequences for self-worth are less affected by their choices. For higher levels of likelihood precision, reducing prior precision has a non-monotonic effect on self-worth. However, when likelihood precision is low, increasing prior precision leads to an *increase* in self-worth. In other words, the effect of likelihood and prior precision show a strong interaction, where the likelihood precision permits a reversal of the effect of prior precision, thereby enabling uncharitable behavior and paradoxically preserved self-worth despite socially inappropriate behavior (i.e., *lacks remorse*). This interaction is entirely consistent with the notion that psychopathy can emerge successively by a reduction sensory likelihood precision (i.e., a failure to attend to prosocial cues), followed by an increase in the precision of prior beliefs (i.e., reduced sensitivity to choices), modeling *lacks remorse* and *self-aggrandizing*, respectively. This interpretation is depicted graphically by the red line in Figure 5.

These results are presented to illustrate that psychopathic behavior—and false inference about the self—can emerge under fairly elementary generative models of one’s own behavior by, and only by, changing the precision of beliefs at different levels of a self-model. Crucially, at no point did we need to change the (simulated) preferences for being approved of (or being rich). Furthermore, the pathological behavior was evident even though all the (synthetic) subjects had exactly the same form of beliefs about the effect of charitable donation on their self-worth. In short, a sufficient explanation for psychopathy (in this minimal example) was a loss of subjective precision linking latent states to observable consequences and an increase in the precision or confidence about the volatility of latent states. The first attenuation—of likelihood precision—means that (synthetic) psychopathic patients can, effectively, ignore (i.e., defend against) evidence that speaks against being the sort of person they would prefer to be (e.g., shame, worthlessness). In a complementary fashion, an increase in the precision of prior beliefs—about self-worth—means that they are more resistant to change and can maintain their self-worth, even in the face of evidence to the contrary. In short, this simple simulation illustrates the profound effect of precision on inference about latent states of the (prosocial) world and, crucially, one’s relationship to that world.

### Psychopathic States and Traits

With this framework, we can also see how the mechanisms underlying *lacks remorse* may operate together with the mechanisms underlying *self-aggrandizing* to defend against shame and worthlessness despite breaching social norms. Specifically, patients can decrease the precision of the bottom-up affective signals from the IWM while simultaneously increasing the precision of the elevated top-down self-appraisal from the high-level prior beliefs (i.e., intermediate beliefs) about the self. This type of defensive functioning appears to closely capture the *self-justifying* trait of psychopathy, whereby patients use rationalization to minimize/deny responsibility for their actions through self-serving (but inaccurate) explanations that preserve their grandiose self-image (Cooke et al., 2004; Perry et al., 2013).

These pathogenic mechanisms may become *entrenched* through a number of processes such that they become stable *traits* over time. The literature on how psychopathic traits develop during childhood and adolescence is still emerging (Frick et al., 2014), and it is clear that all personality pathologies arise from a combination of genetic, temperamental, social, familial, and psychological factors (Alwin et al., 2006). However, *learning* may be a central mechanism underlying the entrenchment of these maladaptive Bayesian inferences. This is because learning is a key mechanism through which the brain structurally and functionally encodes perceived statistical regularities and reinforced associations via experience-dependent synaptic plasticity (Caroni, Donato, & Muller, 2012; Friston, 2010). For instance, the patient may have initially learned this type of defensive functioning from antisocial peers and/or family members who may have been role models during their youth. *Lacks remorse* and *self-aggrandizing* may become entrenched over time through *positive reinforcement* (e.g., from a social milieu that rewards psychopathic personality traits by offering a sense of belonging, support, and self-worth) and *negative reinforcement* (e.g., from the fact that these defense mechanisms relieve the patient from their feelings of worthlessness and shame). Furthermore, the patient’s internal working (i.e., generative) model of the self may also become entrenched over time through patterns of relationships the patient may have experienced from childhood into adulthood. For example, repeated rejecting, punitive, invalidating, and unempathic interactions with peers, teachers, social workers, police and correctional officers, medical and mental health professionals, and even society as a whole play directly into the patient’s core self-image that they are worthless and shameful. This hypothesis regarding the entrenchment of these mechanisms is highly consistent with the CBT model of PDs, which postulates that learningis the process through which coping strategies become fixed, inflexible, and over-/underdeveloped in patients with PDs (Davidson, 2007). In light of the above, what is the evidence that this pathology of inference underlies psychopathy?

## THE NEUROBIOLOGY OF PSYCHOPATHY

In this section, we examine how the Bayesian model of *lacks remorse* and *self-aggrandizing* is supported by existing structural and functional neuroimaging studies on psychopathy in adult samples of highly psychopathic individuals. This section focuses on neuroimaging studies, because neuroimaging is one of the most direct (noninvasive) methods for examining the neurobiology of psychopathology in humans. While there are some core neural abnormalities across studies, it has been noted in prior reviews that some findings are variable (Del Casale et al., 2015; Koenigs, 2012; Koenigs, Baskin-Sommers, Zeier, & Newman, 2011; Seara-Cardoso & Viding, 2015). This variability is likely due to factors such as differences in sample sizes, paradigms, and comparison groups. The sample sizes of some studies of highly psychopathic individuals are comparatively small. Furthermore, neural processing abnormalities observed in psychopathy are at times influenced by the paradigm researchers have used (e.g., a region may be hypo- or hyperactive depending on the task and/or stimuli). Some studies, however, do not use equivalent paradigms or consistent criteria to identify “psychopaths,” which makes it more difficult to compare across samples. Some studies also use different comparison groups (e.g., healthy controls, offenders with low-levels of psychopathic traits) and/or do not match groups on relevant confounding variables (e.g., IQ). Aggregated trait scores (e.g., PCL–R total, factor and facet scores) are typically used to identify and compare groups and/or correlate symptom severity with neural abnormalities, which limits our ability to link neural abnormalities to specific personality *traits*. Finally, the number of neuroimaging studies of psychopathy performed to date is small compared to other forms of psychopathology (e.g., schizophrenia, depression), and thus inferences from the extant neuroimaging data should be considered tentative and will require further empirical investigation (Koenigs et al., 2011).

With these qualifications in mind, it is also important to note that the field has grown immensely in recent years and there are some highly suggestive neuronal abnormalities associated with psychopathy. Crucially, these findings are consistent with the Bayesian model of *lacks remorse* and *self-aggrandizing*. Reviews of the structural MRI, resting-state functional connectivity MRI (rs-fcMRI) and task-based fMRI (t-fMRI) studies of patients with psychopathy suggest that a number of neural networks involving frontal, limbic, and paralimbic structures are disturbed in psychopathy (N. E. Anderson & Kiehl, 2012; Blair, 2007, 2008, 2010, 2013; Del Casale et al., 2015; Kiehl, 2006; Koenigs, 2012; Koenigs et al., 2011; Seara-Cardoso & Viding, 2015). We focus on neural network abnormalities in the amygdala–ventromedial prefrontal cortex (vmPFC)^4^ network. It is important to note at the outset that Blair (2007, 2008, 2010, 2013) has argued for many years that a core neural abnormality characterizing psychopathy involves dysfunction along the amygdala–vmPFC network. Thus the neurobiological implementation of the Bayesian model we outline in what follows is consistent with the framework developed by Blair (2007, 2008, 2010, 2013).

### Review of Neuroimaging Research

Diffusion tensor imaging (DTI) studies of psychopaths provide a window into the abnormalities in the amygdala–vmPFC network. DTI allows researchers to reconstruct in vivo the white matter (WM) tracts connecting brain regions and to evaluate certain aspects of its microstructure. Six DTI studies comparing psychopaths with matched controls found reduced WM structural integrity in the uncinate fasciculus (Craig et al., 2009; Hoppenbrouwers et al., 2013; Jiang et al., 2017; Motzkin, Newman, Kiehl, & Koenigs, 2011; Sundram et al., 2012; Waller, Dotterer, Murray, Maxwell, & Hyde, 2017; Wolf et al., 2015). The uncinate fasciculus is a bidirectional hook-shaped tract with fibers that connect the temporal pole (TP; Brodmann area [BA] 38/20), entorhinal cortex (ERC; BA 28/34), perirhinal cortex (PRC; BA 35/36), parahippocampal cortex (PHC; BA 36), and amygdala to regions of the lateral orbitofrontal cortex (lOFC; BA 11/47), vmPFC (BA 11/32), and rostromedial PFC (rmPFC; BA 10; Schmahmann & Pandya, 2006; Schmahmann et al., 2007; Thiebaut de Schotten, Dell’Acqua, Valabregue, & Catani, 2012; Von Der Heide, Skipper, Klobusicky, & Olson, 2013). The uncinate fasciculus fibers split when they enter the frontal region into a larger ventro-lateral branch and a smaller anterior-medial branch (Catani, Howard, Pajevic, & Jones, 2002). The ventro-lateral branch terminates in the lOFC, whereas the antero-medial branch terminates in rmPFC. Crucially, rs-fcMRI studies of psychopaths mirror this structural connectivity deficit: psychopaths also show reduced functional connectivity between the amygdala and vmPFC at rest (Motzkin et al., 2011), while passively watching scenes depicting moral violations (Yoder, Harenski, Kiehl, & Decety, 2015), and while imagining others in pain (Decety, Chen, Harenski, & Kiehl, 2013) compared to age, gender, and IQ matched controls, though one study found that this de-coupling was more dorsal in the mPFC (Contreras-Rodríguez et al., 2015). Furthermore, structural brain changes associated with psychopathy overlap with the brain regions connected by the uncinate fasciculus. For instance, psychopathy is associated with significant reductions in gray matter volume (GMV), gray matter density (GMD), and/or cortical thickness in the rmPFC, vmPFC, lOFC, amygdala,^5^ TP, PHC, ERC, and PRC (Boccardi et al., 2011; Contreras-Rodríguez et al., 2015; de Oliveira-Souza et al., 2008; Ermer, Cope, Nyalakanti, Calhoun, & Kiehl, 2012; Gregory et al., 2012; Ly et al., 2012; Müller et al., 2008; Yang, Raine, Colletti, Toga, & Narr, 2009, 2010; Yang, Raine, Narr, Colleti & Toga, 2009).

By linking these abnormalities to the amygdala–vmPFC network’s normal functions, we can understand the significance of these results in relation to the Bayesian model described in the section “A Bayesian Account of Psychopathy.” The amygdala is a complex collection of nuclei with extensive connections with cortical and subcortical structures. The amygdala receives inputs from all sensory modalities and has mainly unidirectional output projections to the striatum and rich bidirectional connections with the mPFC, OFC, medial temporal lobe (MTL; i.e., ERC, PRC, PHC, hippocampus), TP, thalamus, hypothalamus, and brain stem (Freese & Amaral, 2005; Ghashghaei & Barbas, 2002; Ghashghaei, Hilgetag, & Barbas, 2007; Janak & Tye, 2015; McDonald, 1998; Sah, Faber, De Armentia, & Power, 2003). Early accounts of the amygdala linked it principally to fear-related processing; however, a great amount of evidence now suggests that this is a simplification, for the amygdala is involved in affective processing, social behavior, and reward learning. What appears to unify these functions is that the amygdala provides a basic signal of the “salience,” “affective significance” or “value” of sensory information and semantic/episodic/autobiographical memory representations stored in the TP and MTL (Binder & Desai, 2011; Martinelli, Sperduti, & Piolino, 2013; Olson, McCoy, Klobusicky, & Ross, 2013; Squire, Stark, & Clark, 2004), and this information is then transmitted to higher-cortical areas, particularly the vmPFC and lOFC, for hierarchically deeper or more elaborate processing (Adolphs, 2010; Balleine & Killcross, 2006; Baxter & Murray, 2002; Janak & Tye, 2015; Morrison & Salzman, 2010; Murray, 2007). In the context of active inference, salient information that is transmitted to higher levels in the hierarchy corresponds to information that is afforded greater precision. Similarly, the “rewarding” aspects of a cue entail greater precision or confidence about the consequences of action (Friston, Schwartenbeck et al., 2014). We will therefore use *salience*, *reward*, and *precision* interchangeably in this article.

DTI and neuropsychological research has strongly suggested that this transmission of affective salience-tagged (i.e., high precision) mnemonic and sensory information to the vmPFC/lOFC is one of the core functions of the uncinate fasciculus (Von Der Heide et al., 2013). The bidirectional nature of the uncinate fasciculus means that the vmPFC/lOFC can also modify the affective significance of these representations (e.g., by furnishing prior biases or predictions), which is consistent with the fact that the vmPFC is implicated in emotion regulation and fear extinction (Etkin, Egner, & Kalisch, 2011; Schiller & Delgado, 2010).

The vmPFC and lOFC therefore play a central role in this network. Their different patterns of anatomical connectivity with cortical and subcortical structures provide clues concerning their different functions (Bandler, Keay, Floyd, & Price, 2000; Barbas & Pandya, 1989; Carmichael & Price, 1995a, 1995b; Haber, 2016; Noonan, Kolling, Walton, & Rushworth, 2012; Öngür & Price, 2000; Price, 2007; Rudebeck & Murray, 2011; Saleem, Kondo, & Price, 2008; Saleem, Miller, & Price, 2014; Wallis, 2007). The vmPFC and lOFC are densely interconnected with each other and have bidirectional connections with the amygdala, hippocampus, ERC, PRC, PHC, TP, mediodorsal thalamus (MD), and lateral PFC. However, there are a number of notable differences: The vmPFC has bidirectional connections with the posterior cingulate cortex (PCC) and retrosplenial cortex (Rsp), which is not apparent in the lOFC. Furthermore, the lOFC receives inputs from visual, olfactory, gustatory, and somatosensory modalities, whereas the vmPFC receives relatively few direct sensory inputs. These anatomical differences are consistent with the fact that the vmPFC is a central hub in the *default mode network* (DMN)^6^ and thus plays a central role in *internal mentation* and *self-related processing* (Andrews-Hanna, 2012; Martinelli et al., 2013; Northoff et al., 2006; Qin & Northoff, 2011). Finally, although both regions project to the striatum (primarily the ventral striatum), only the vmPFC sends outputs to the hypothalamus and periaqueductal gray (PAG). This allows the vmPFC to potentially modulate a wide range of basic physiological functions mediated by the hypothalamus and PAG: pain modulation, fear/anxiety, stress response, fight–flight response, sleep–wake cycle, sexual/parental behaviors, energy metabolism, body temperature, blood pressure/electrolyte composition, and the autonomic nervous system (Benarroch, 2012; Kandel, Schwartz, Jessell, Siegelbaum, & Hudspeth, 2013). In terms of the Bayesian brain, this functional anatomy is associated with interoceptive inference and providing predictions that engage autonomic reflexes that are essential for affiliative, prosocial, and other actions (Barrett & Simmons, 2015; Pezzulo, Rigoli, & Friston, 2015; Seth, 2015; Seth & Friston, 2016).

While the lOFC and vmPFC are both implicated in value-based processing and decision-making, research suggests that their functions are distinct (for reviews, see Kable & Glimcher, 2009; Levy & Glimcher, 2012; Noonan et al., 2012; Peters & Büchel, 2010; Rangel & Hare, 2010; Rudebeck & Murray, 2011; Sescousse, Caldú, Segura, & Dreher, 2013; Stalnaker, Cooch, & Schoenbaum, 2015). Although there are still many unanswered questions, the lOFC appears to be involved in learning and assigning value/salience to specific stimuli based on their association with specific reward outcomes, whereas the vmPFC encodes the *expected subjective value* of various stimuli into a continuous “common currency” to determine which is most salient/significant/precise. Specifically, the vmPFC encodes the expected values of various types of information, which can be applied not only to stimuli from the *external environment*, but also *internally generated*, *self-related mental contents* in order represent the personal significance/salience of this information along a continuum (for a review, see D’Argembeau, 2013). The encoding of the expected value of internal/external stimuli (i.e., “valuation”) by the vmPFC is believed to not only facilitate the comparison among options in order to choose the most significant/salient option during decision-making (Kable & Glimcher, 2009; Levy & Glimcher, 2012; Peters & Büchel, 2010), but it is also hypothesized that valuation is essential for constructing a coherent self-representation (D’Argembeau, 2013).

The anatomical connectivity of the lOFC/vmPFC is consistent with this functional differentiation (Rudebeck & Murray, 2011). Specifically, the connections between the lOFC and visual, olfactory, gustatory, and somatosensory regions, as well as amygdala and MTL, means that the lOFC is in an ideal anatomical position to construct a high-dimensional representation of the values of specific stimuli using information about their sensory, memory, and affective components. Similarly, the connections between vmPFC and amygdala, MTL, and lOFC mean that the vmPFC is in an ideal anatomical position to integrate multiple value signals into a one-dimensional representation (“common currency”) of expected value (e.g., the complex value representations from the lOFC and the more basic salience-tagged memory representations and sensory information conveyed along the uncinate fasciculus). Finally, it has been hypothesized that the lateral PFC has complex interactions with the lOFC and vmPFC (Kable & Glimcher, 2009; Northoff et al., 2006; Rangel & Hare, 2010; Wallis, 2007). Specifically, the lateral PFC may exert top-down modulation of the value-based processes performed by the lOFC/vmPFC, selecting and filtering among the set of options generated by the vmPFC/lOFC.

**Figure 6.**
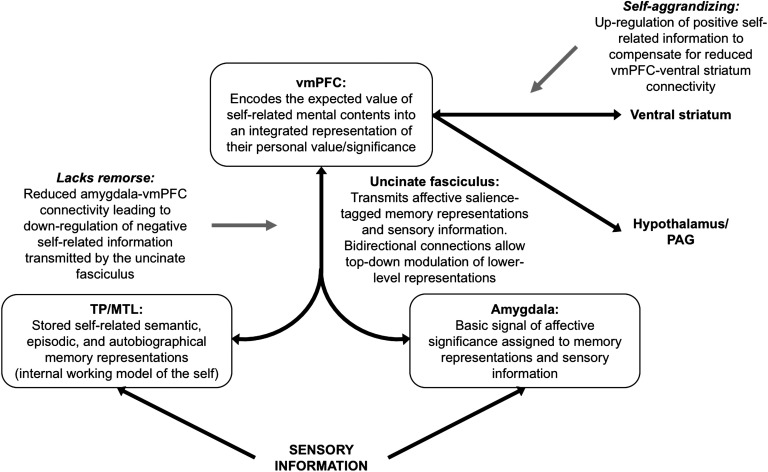
**A neurobiological implementation of the Bayesian model of *lacks remorse* and *self-aggrandizing*.** The internal working model (IWM) of the self consists of semantic, episodic, and autobiographical memory representations stored in the TP/MTL. Self-related sensory information from the external environment and social interactions are interpreted through the representations of the IWM of the self. The amygdala provides a basic signal of affective significance to the self-related sensory/mnemonic information stored in the TP/MTL. The amygdala thus encodes affective and interoceptive expectations that contextualize this information. The self-related information in the TP/MTL is transmitted to the vmPFC along the uncinate fasciculus. The vmPFC integrates these signals with ascending prediction errors from the amygdala to update an integrated representation of the expected value of the self-related information it is receiving. The vmPFC self-representation may then modulate hypothalamic and PAG activity to elicit basic physiological responses (e.g., via autonomic reflexes). Reduced amygdala–vmPFC connectivity leads to *lacks remorse*, attenuating the negative affective and interoceptive information transmitted along the uncinate fasciculus. *Self-aggrandizing* reflects the augmentation of positive self-related information in response to the reduced vmPFC–ventral striatum connectivity, which impairs the integration of self-representations with signals of positive affect and reward mediated by the ventral striatum. See main text for details. TP = temporal pole; MTL = medial temporal lobe; PAG = periaqueductal gray; vmPFC = ventromedial prefrontal cortex.

As illustrated in Figure 6, the amygdala–vmPFC network provides a possible neurobiological implementation of the computational architecture underlying *lacks remorse* and *self-aggrandizing* described in the section “A Bayesian Account of Psychopathy.” In this model, the trait of *lacks remorse* reflects a tendency to down-regulate negative self-related processing (e.g., feelings of shame/worthlessness), which we propose would be associated with diminished connectivity between the amygdala and vmPFC along the uncinate fasciculus. In predictive coding terms, this would correspond to an attenuation of (the precision of) prediction errors ascending from the amygdala that would normally inform and update self-representations in the vmPFC. These representations (i.e., expectations) would normally integrate affective, prosocial, and interoceptive cues with those based on representations encoded in the MTL through the assimilation of ascending prediction errors conveyed by ascending connections in the uncinate fasciculus.

While no studies have directly correlated trait-level facets of psychopathy with functional connectivity patterns, studies have found that psychopathy in general is associated with reduced functional connectivity between the amygdala and vmPFC (Decety, Chen et al., 2013; Motzkin et al., 2011; Yoder et al., 2015). Over time, this functional de-coupling could lead to a structural de-coupling, which is consistent with DTI studies reporting that psychopathy is associated with reduced WM structural integrity in the uncinate fasciculus (Craig et al., 2009; Hoppenbrouwers et al., 2013; Jiang et al., 2017; Motzkin et al., 2011; Sundram et al., 2012; Waller et al., 2017; Wolf et al., 2015). Accordingly, this model predicts that a person’s score for *lacks remorse* should be inversely correlated to the strength of amygdala–vmPFC connectivity. Furthermore, this de-coupling between the amygdala and vmPFC is consistent with numerous t-fMRI studies which have demonstrated that psychopathy is associated with less activation in these structures in response to negative stimuli. For instance, psychopaths show reduced activation of the amygdala and/or vmPFC while perceiving others’ pain (Decety, Skelly, & Kiehl, 2013), imagining others in pain (Decety, Chen et al., 2013), viewing others’ facial expressions of fear, sadness, and pain (Decety, Skelly, Yoder, & Kiehl, 2014; Dolan & Fullam, 2009), during a recognition memory task for negative affective words (Kiehl et al., 2001), during aversive conditioning (Birbaumer et al., 2005; Schneider et al., 2000; Veit et al., 2002), and during emotional moral processing (Fede et al., 2016; Glenn, Raine, & Schug, 2009; Harenski, Harenski, Shane, & Kiehl, 2010; Yoder et al., 2015). The trait *lacks remorse* can therefore be understood as emerging from the increasing functional and structural de-coupling between the amygdala and vmPFC, which attenuates the negative affective salience-tagged self-related memory representations and sensory information (i.e., prediction errors) transmitted along the uncinate fasciculus. This attenuation or de-coupling effectively cuts off the vmPFC from integrating affective information into the representation the vmPFC is constructing of the expected value of self-related mental constructs (i.e., posterior expectations). This is highly consistent with the Bayesian formulation of *lacks remorse* described earlier, which characterizes this personality trait as an attenuation of the precision of the ascending prediction errors arising from the patient’s IWM, which decreases the influence of negative affective inputs on the patient’s posterior expectations about their self (Figure 3). In this way, we can understand the possible neurobiological implementation of the Bayesian architecture underlying *lacks remorse*.

Given that the vmPFC modulates activity in the hypothalamus and PAG (Figure 6), this may also explain well-established physiological findings associated with psychopathy. Meta-analytic evidence shows that psychopathy is associated with significantly lower electrodermal activity at rest, during a task, in response to negative stimuli (e.g., anger provoking, painful, or aversive stimuli), and as a change from baseline (Lorber, 2004). This blunting of sympathetic nervous system (SNS) activity is consistent with the neurobiology of psychopathy and the attenuation of affectively charged prediction errors (see Stephan et al., 2016, for a related discussion of interoceptive inference in the context of fatigue and depression). Specifically, one would predict from the amygdala-vmPFC de-coupling associated with psychopathy that the basic affective signals transmitted along the uncinate fasciculus would not only fail to influence the valuation processes in the vmPFC, but also brain regions modulated by the vmPFC, such as the hypothalamus and PAG, which can regulate SNS activity (Benarroch, 2012; Kandel et al., 2013; Seth & Friston, 2016; Stephan et al., 2016). Such physiological findings are consistent with the hypothesis put toward in this article that *lacks remorse* is generated by a discounting of affective information, which is mediated by amygdala-vmPFC de-coupling along the uncinate fasciculus.

Although more speculative, there is reason to believe that *self-aggrandizing* may be associated with a frontostriatal circuit involving the vmPFC and ventral striatum. In support of this proposal, there is evidence that self-esteem is related to frontostriatal connectivity. Specifically, *trait* self-esteem is related to increased WM structural integrity between ventral striatum and mPFC (including vmPFC), whereas *state* self-esteem is related to increased functional connectivity along this circuit (Chavez & Heatherton, 2015). The authors of this study hypothesized that frontostriatal connectivity may reflect an integration of self-representations encoded in the vmPFC with positive affect and reward/precision signals mediated by the ventral striatum (Haber, 2016). Interestingly, the “superiority illusion”—the cognitive bias reflected in people tending to evaluate themselves as superior to average—is associated with resting-state functional connectivity between the mPFC and striatum, which was found to be regulated by inhibitory dopaminergic neurotransmission (Yamada et al., 2013). Most relevant to *self-aggrandizing* is the finding that grandiose narcissism is associated with *reduced* frontostriatal WM structural integrity between the vmPFC and ventral striatum (Chester, Lynam, Powell, & DeWall, 2016), which is the opposite of individuals with high trait self-esteem (Chavez & Heatherton, 2015). The authors of this study hypothesized that grandiosity may reflect a mechanism that compensates for this neural deficit in the circuitry that connects the brain’s reward systems (i.e., sources of neuromodulatory precision setting projections) with its self-representations, such that grandiose individuals have a low “baseline” self-reward connectivity. The neural regions involved in compensating for this low baseline by abnormally increasing a person’s self-appraisal are unknown and likely involve multiple interacting systems. One likely neural system is the lateral PFC, which can influence valuation and self-referential processes in the vmPFC via top-down modulation (Kable & Glimcher, 2009; Northoff et al., 2006; Rangel & Hare, 2010; Wallis, 2007). While more research is needed, the emerging neuroimaging evidence on grandiosity is consistent with the Bayesian model of *self-aggrandizing* described earlier, which characterizes this trait as compensatory prior beliefs (i.e., intermediate beliefs) about the self (i.e., a defense mechanism), which upregulates positive self-related information in the face of low baseline self-worth (e.g., feelings of shame/worthlessness), resulting in abnormally elevated self-appraisal.

### Putting It All Together

Taken together, while the neuroimaging literature to date is small, the computational architecture depicted in Figure 3 can be mapped onto the neural network associated with psychopathy depicted in Figure 6. The vmPFC corresponds approximately to the intermediate level of the architecture (Figure 3), which encodes the conscious posterior expectations about the self, which is generated by integrating descending top-down prior predictions about the self with ascending prediction error signals from regions supplying other information about the self. This hypothesis that the vmPFC plays a central role in the automatic conscious thoughts about the self in psychopathy is in keeping with the fact that the vmPFC is a central hub in large-scale brain networks that mediate internal mentation and self-related processing (Andrews-Hanna, 2012; Martinelli et al., 2013; Northoff et al., 2006; Qin & Northoff, 2011). The neural regions underlying the high-level prior beliefs encoded in the top level of Figure 3 are currently unknown, though the lateral PFC is likely a key neural system mediating these top-down predictions by virtue of the fact that it can exert conscious top-down modulation of valuation and self-referential processes in the vmPFC (Kable & Glimcher, 2009; Northoff et al., 2006; Rangel & Hare, 2010; Wallis, 2007). The IWM of the self consists of semantic, episodic, and autobiographical memory representations stored in the TP/MTL, with the amygdala providing a basic signal of affective significance to the self-related information stored in these regions. This self-related information is transmitted to the vmPFC along the uncinate fasciculus. Thus the uncinate fasciculus corresponds approximately to the ascending connections to the intermediate layer, and the TP/MTL–amygdala network corresponds approximately to the bottom level of the network representing the contents of the IWM of the self (Figure 3).

It is for this reason that we hypothesize that *lacks remorse* is mediated by functional and structural de-coupling between the amygdala and vmPFC along the uncinate fasciculus, which effectively cuts off the vmPFC from integrating information from lower levels in the hierarchy (i.e., the IWM of the self) into the posterior expectations it is forming about the self. Given the present state of affairs on the neuroimaging of psychopathy, it is more difficult to make such close links between the computational architecture underlying *self-aggrandizing* and the neural networks associated with psychopathy. Recall that Figure 3 hypothesizes that *self-aggrandizing* is mediated by top-down predictions supplied by priors from higher levels in the hierarchy, which modulate the conscious posterior expectations of the self encoded at the intermediate level. As outlined in the preceding review, this specific hypothesis has not yet been tested in the neuroimaging literature. This is because there are no studies to date specifically investigating the link between psychopathy and the brain regions that likely mediate such top-down predictions on the vmPFC during self-related processing (e.g., lateral PFC). That said, the literature to date has suggested that frontostriatal dysconnectivity, which normally connects the brain’s conscious self-representations in the vmPFC with the brain’s reward systems, may contribute to the negative core self-image characteristic of patients with the trait *self-aggrandizing*. This is compensated for via other brain regions, which we hypothesize the lateral PFC being key among them.

Therefore, based on the emerging neuroimaging research reviewed earlier, it appears that there are at least two, potentially overlapping, pathways that mediate the “undefended” negative core self-image underlying *lacks remorse* and *self-aggrandizing* (Figure 3A). The first is the amygdala–vmPFC connectivity along the uncinate fasciculus. The second is the frontostriatal dysconnectivity. While there are at least two pathways, the net effect on self-representations in the vmPFC appears to be the same. Specifically, in the absence of a compensatory de-coupling between the amygdala–vmPFC network (i.e., an undefended state), the psychopathic patient’s amygdala–vmPFC connectivity increases the likelihood that vmPFC self-representations will be influenced by the negative affective salience-tagged self-related memory representations and sensory information transmitted along the uncinate fasciculus. Similarly, in the absence of a compensatory up-regulation of self-related information in the vmPFC (i.e., an undefended state), the psychopathic patient’s frontostriatal dysconnectivity increases the likelihood that vmPFC self-representations will not be integrated with positive affective and reward signals mediated by the ventral striatum. Thus, in both cases, vmPFC self-representations are characterized by negative affective predictions in the absence of compensatory (defense) mechanisms, by virtue of either the presence of negative affective signals from lower level brain regions (i.e., amygdala–vmPFC connectivity) or the absence of positive affective/reward signals from the ventral striatum (i.e., frontostriatal dysconnectivity).

One unanswered question is whether or not these pathways are present in both *lacks remorse* and *self-aggrandizing*, or whether they represent different neurobiological instantiations of the computational architecture outlined in Figure 3. That is to say, the undefended state in *lacks remorse* and *self-aggrandizing* could be mediated both by amygdala–vmPFC connectivity and frontostriatal dysconnectivity, or the pathways may be unique to each trait (e.g., the negative core self-image in *self-aggrandizing* may be mediated uniquely by frontostriatal dysconnectivity, whereas the negative core self-image in *lacks remorse* may be mediated uniquely by amygdala–vmPFC connectivity). Relatedly, it may be that *self-aggrandizing* and *lacks remorse* are both associated with defensive de-coupling between the amygdala–vmPFC along the uncinate fasciculus. This would be consistent with our model, given that both are defensive responses to negative self-related information. However, the present state of psychopathy neuroimaging research does not provide firm answers to these questions. This is because there has not yet been an analysis of structural or functional neuroimaging data examining the relationship between the traits *lacks remorse* and *self-aggrandizing* and these two pathways in the same sample of psychopathic patients. Such a study would help shed light on these hypotheses regarding the neurobiological instantiation of the undefended negative core self-image underlying *lacks remorse* and *self-aggrandizing* and the neural circuitry of these defense mechanisms.

## DISCUSSION

While by no means the last word on these topics, we believe that the computational architecture (Figure 3), quantitative simulations (Figures 4 and 5), and neuroimaging research on psychopathy (Figure 6) provide a working formal model of *lacks remorse* and *self-aggrandizing* as a form of abnormal Bayesian inference about the self. Furthermore, we believe that this formal model provides a computational neuroscientific basis for the integrated psychotherapeutic model of these traits which cognitive and psychodynamic theories have converged on (see the section “Toward an Integrated Psychotherapeutic Model of Psychopathy”). That being said, there are a number of limitations of the model we have proposed, some of which are intrinsic to the model outlined herein, and others are a consequence of the limitations in the current state of affairs in psychopathy research.

The model is limited, first, because it has provided an explanatory framework only for traits *lacks remorse* and *self-aggrandizing*. However, there are many other psychopathic traits (Table 1), and these require an explanation in terms of Bayesian inference for this model to be a sufficiently comprehensive model of psychopathy. Second, the model has not specified the precise neuromodulatory mechanisms controlling the precision of prior beliefs and bottom-up evidence (from the IWM of the self) on automatic thoughts about the self. Furthermore, as mentioned, there is uncertainty regarding the neural correlates of the high-level prior beliefs that up-regulate self-related information in response to frontostriatal dysconnectivity. This means that we do not yet have a direct mapping of this component of the computational architecture (Figure 3) onto the neurobiology of psychopathy, though there are likely candidate neural systems (i.e., the lateral PFC). Similarly, as described earlier, it is at present uncertain whether frontostriatal dysconnectivity is unique to *self-aggrandizing*, or whether it also contributes to the negative affective self-image in *lacks remorse*.

There are other limitations that stem more from limitations inherent in the current state of affairs in psychopathy research. Chief among them is the fact that the neuroimaging literature on psychopathy to date is small compared to other forms of psychopathology, and thus inferences from this literature should be considered tentative and will require further confirmation (Koenigs et al., 2011). Along the same lines, we know very little about the neurochemical basis of the psychopathic traits covered in this article. There are at least two reasons for this. First, psychopathy, and PDs more generally, are extremely complex clinical phenomena, and there are currently no plausible translational animal models of psychopathy, especially the core *Antagonism* traits. While there are undoubtedly animal models of *Disinhibition* traits (e.g., impulsivity), these are not the core features of the psychopathic personality (Poythress & Hall, 2011; Skeem & Cooke, 2010). In the absence of plausible translational models, PET imaging is the gold standard for examining in vivo neurochemical disturbances associated with psychiatric diseases in humans. Unfortunately, to the best of our knowledge, there is only one PET imaging study to date using an adult sample of highly psychopathic individuals, however, this study focused specifically on impulsivity and not *Antagonism* traits (Kolla et al., 2015). This is the second reason for our limited understanding of the neuromodulatory mechanisms underlying psychopathy. In short, we simply do not know as much about the neurobiological basis of psychopathic traits as we do about, for example, schizophrenia or depression. This places an upper limit on our ability to provide a detailed model of the neurobiological circuitry underlying *lacks remorse* and *self-aggrandizing*. Finally, another major limitation in the field is that aggregated trait scores (e.g., PCL–R total, factor and facet scores) are typically used in neuroimaging studies. In our view, this is a significant methodological and analytic problem. This is because it limits the field’s ability to link neural abnormalities to specific psychopathic traits. It is for this reason that we had to make indirect links between the neuroimaging findings and the specific traits of *lacks remorse* and *self-aggrandizing*. Therefore, the field would benefit immensely from either new neuroimaging studies that analyze the data at the trait-level, or even reanalyses of older datasets to investigate the associations between specific psychopathic traits and brain structure/function.

### Treatment Implications: Integrated Modular Treatment for Psychopathy

A full discussion of the treatment implications of the Bayesian model, and the integrated psychotherapeutic model more generally, extends beyond the scope of this article. That being said, an outline of the treatment implications is possible. The etiological framework of our Bayesian model resonates with major trends in how some clinicians are beginning to conceptualize the treatment of PDs. Over the past number of years, there has been a growing appreciation that specialized therapies rooted in specific “schools” of psychotherapy (e.g., CBT vs. SFT vs. dialectical behavior therapy vs. transference-focused psychotherapy vs. mentalization-based treatment) tailored to specific diagnostic categories may not be the most effective strategy for treating PD. Rather, it may be more effective to utilize an integrated approach to PD treatment. It is for this reason that the *integrated modular treatment* (IMT) was developed (Clarkin, Cain, & Livesley, 2015; Livesley, 2003, 2005, 2007a, 2007b, 2012; Livesley, Dimaggio, & Clarkin, 2016). Our model resonates closely with the IMT and its integrated approach to PD treatment. Before summarizing the IMT and how our model fits into this framework, it is necessary to outline the three major reasons for shifting toward an integrated approach to PD treatment: (a) the evidence for “common factors,” (b) the utility of technical eclecticism, and (c) the evidence for theoretical integration across schools of psychotherapy (Livesley et al., 2016).

The first reason is the evidence for *common factors* across therapies. This is the finding that there are few clinically significant differences in the efficacy across psychotherapies for PD, including general psychiatric management and supportive psychotherapy (Budge et al., 2013; Clarkin, Levy, Lenzenweger, & Kernberg, 2007; Cristea et al., 2017; Leichsenring & Leibing, 2003; McMain et al., 2009). Although there are sometimes differences in outcome between studies, they are often small and/or difficult to interpret because they rarely demonstrate clear superiority of one specialized therapy from a particular “school” over another in head-to-head comparisons. The lack of evidence for clinically significant differences in outcome across therapies is seen in studies of treatments for borderline PD (Cristea et al., 2017) and antisociality (Landenberger & Lipsey, 2005; Lipsey, Landenberger, & Wilson, 2007). Of course, this is not surprising at all. It was pointed out as early as the 1930s by Rosenzweig that there are a set of “common factors” that underlie all bona fide psychotherapies, and these *general change mechanisms* common to all therapies for PD explain the equivalent efficacy across treatments (Rosenzweig, 1936). Indeed, there is now compelling evidence from the general psychotherapy literature that there is a set of common factors shared across specialized therapies that together account for a large proportion (at least 50%) of the variance in positive outcome (Horvath & Symonds, 1991; Luborsky, Singer, & Luborsky, 1975; Luborsky et al., 2002; Marcus, O’Connell, Norris, & Sawaqdeh, 2014; Martin, Garske, & Davis, 2000; Wampold, 2001). What are these general change mechanisms? Lists of these common factors sometimes differ; however, the common factors can be grouped into six general change mechanisms (Livesley et al., 2016):

1.  *Structure.* Establish a highly structured treatment process that defines the *therapeutic stance* (i.e., therapist provides support, empathy, and validation) and establishes an explicit *treatment contract* that defines the purpose, format, terms, and limits (e.g., treatment boundaries) of the therapy.
2.  *Treatment alliance.* Establish and maintain a collaborative treatment alliance.
3.  *Consistency.* Maintain a consistent treatment process by adhering to the treatment structure (i.e., the therapeutic stance and treatment contract).
4.  *Validation.* The therapist promotes validation by recognizing and affirming the legitimacy of the patient’s experience (i.e., that the patient’s thoughts, feelings, and behavior make sense and are understandable).
5.  *Motivation.* Build motivation and commitment to change.
6.  *Meta-cognition.* Promote self-observation, self-knowledge, and self-reflection.

In commenting on the causal role of common factors in psychotherapy, Markowitz (2014) has underscored the following paradox: “*if you cannot do this* [i.e., deliver the common factors], *the rest of psychotherapy does not matter*”; however, “*if you can do this, the rest of psychotherapy does not matter* [because the majority of the causal ingredients linked to positive outcome are provided by these general change mechanisms]” (pp. 287–288, emphasis original).

The evidence for common factors, however, does not preclude the possibility that specific interventions unique to specialized therapies may contribute to positive outcomes in PD, independent of general change mechanisms. In other words, there may also be *specific change mechanisms* required for specific types of dysfunction, and thus specific interventions may be required for the unique problems seen in particular patients. Those therapies that contain these “specific ingredients” (i.e., specific interventions) will show superior efficacy to those that do not. The empirical literature suggests that this may indeed be the case. Meta-analytic evidence suggests that acute symptoms may respond better to more structured cognitive-behavioral techniques than less structured psychodynamic techniques (Marcus et al., 2014). Conversely, psychodynamic techniques may be more effective for increasing self-reflection relative to structured cognitive-behavioral techniques (Livesley et al., 2016). Similarly, in the realm of rehabilitation for people involved in the criminal justice system, structured cognitive-behavioral techniques are robustly associated with reduced violent/antisocial behavior, whereas generic interventions are not (Andrews et al., 1990; Landenberger & Lipsey, 2005; Lipsey & Cullen, 2007; Lipsey et al., 2007; McGuire, 2008).

The take home message is that the empirical literature on PD treatment, and psychotherapy more generally, suggests that effective, evidence-based treatment for PD requires an integrative “*both/and*” approach, rather than a tribal “*either/or*” approach that forces clinicians to choose between different specialized treatments from different schools of psychotherapy (Marcus et al., 2014): treatment should *both* explicitly utilize general treatment interventions shared across specialized therapies to maximize common factors *and* explicitly utilize specific treatment techniques that have been shown to be effective for specific types of dysfunction.

This dovetails into the second reason for the IMT approach, which is the utility of *technical eclecticism* over fidelity to the prescribed techniques of specialized treatments (Livesley et al., 2016). This is because specialized treatments often reduce the range of psychopathology seen in PD to a single primary impairment. For example, the primary impairment in borderline PD has been explained in terms of (a) affect dysregulation = dialectical behavior therapy, (b) dysfunctional beliefs = CBT, and (c) deficits in mentalization = mentalization-based therapy (Bateman & Fonagy, 2016; Davidson, 2007; Linehan, 1993). Although simplifying clinical complexity is helpful, the problem is that such reductions narrow the focus of treatment and the selection of interventions to only those implied by the underlying theory (e.g., dialectical behavior therapy focuses on increasing the patient’s affect regulation skills). Specialized treatments thus risk neglecting other explanatory factors and thus other potentially useful techniques to help treat the diverse range of problems patients face (e.g., patients with borderline traits have difficulties with affect regulation but also maladaptive cognitions, mentalization, impulse control, self and interpersonal problems, etc.). Technical eclecticism avoids this theory-imposed restriction by integrating specific interventions from diverse therapies, without necessarily endorsing all their underlying theoretical constructs, in order to address the full range of impairment seen in PD.

The third reason for an integrated approach to PD treatment is that the psychotherapeutic field is converging on a number of core ideas about the etiology of PD. A concrete example of such *theoretical integration* was described in the section “Toward an Integrated Psychotherapeutic Model of Psychopathy,” where we saw the convergence of psychodynamic, CBT, and SFT models of the traits *lacks remorse* and *self-aggrandizing*. While often described using different terminology, almost all current evidence-based psychotherapies for PD emphasize at least some, and sometimes all, of the following: (a) that the maladaptive thoughts, feelings, and behaviors seen in PD are driven by entrenched cognitive–affective structures (e.g., IWM, schemas); (b) that these cognitive–affective structures are often not fully consciously articulated or accessible (i.e., they are unconscious or partially conscious) yet organize and influence a patient’s conscious experiences and behavior; (c) that genetics, learning, developmental factors, and the quality of attachment experiences play central causal roles in shaping these cognitive–affective structures and thus current psychopathology; and (d) that some of the psychopathology observed in patients with PD is a consequence of the operation of maladaptive “defense” or “coping” mechanisms that protect against distressing or unacceptable thoughts and feelings.

The IMT model is an integrated approach to PD treatment and was developed to explicitly address these three issues. The IMT model integrates the treatment principles and methods that work across therapies, called *general treatment modules*, with *specific treatment modules* which consist of an eclectic array of specific interventions, in order to target both the common and unique features, respectively, of patients with PD (Clarkin et al., 2015; Livesley, 2003, 2005, 2007a, 2007b, 2012; Livesley et al., 2016). Thus general treatment modules target the core impairments in self and interpersonal functioning common to all personality pathology (APA, 2013; Bender et al., 2011; Livesley, 2011; Morey et al., 2015; Morey et al., 2011; Skodol, 2012, 2014), whereas specific treatment modules target the unique profile of the patient’s pathological personality traits. More specifically, general treatment modules explicitly utilize the common factors (i.e., structure, treatment alliance, consistency, validation, etc.) in order to create a therapeutic process that provides a *continuous corrective experience* that treats the core impairments in self/interpersonal functioning. Thus general treatment modules are used with all patients and throughout treatment. Conversely, specific treatment modules are selected from all therapies, based on empirical and rational considerations, to treat the specific domains of dysfunction that are the current focus of treatment. In the IMT, the focus of treatment, and thus the selection of specific interventions, is determined by the domains of dysfunction that are currently present in the patient: (a) *acute symptoms* (e.g., dysphoria, rage, self-harm/suicidal behavior, violence), (b) *regulation and modulation* (e.g., difficulty regulating maladaptive thoughts, feelings, and behavior), (c) *interpersonal problems* (e.g., conflictual relationships), and (d) *self problems* (e.g., difficulty regulating self-esteem, unstable and fragmented identity). These four domains of dysfunction imply a hierarchy of treatment foci (i.e., priority is given to specific interventions that target acute symptoms when this domain is present over specific interventions targeting interpersonal problems). Furthermore, they imply that patients typically progress, not necessarily linearly, through five *phases of change* during their treatment with the IMT (Clarkin et al., 2015; Livesley, 2003, 2005, 2007a, 2007b, 2012; Livesley et al., 2016):

*Phase 1 of Change: Safety.* The primary goal is to ensure the safety of self and others (i.e., crisis management). This is done through the general treatment modules of structure, validation, and support, which often successfully ensure safety in a community setting. However, sometimes safety cannot be ensured in the community with general change mechanisms alone—a situation routinely encountered in corrections and forensic mental health settings. This requires supplementing them with specific interventions, including inpatient hospitalization and/or restraints and seclusion.^7^
*Phase 2 of Change: Containment.* The primary goal is to contain affective, behavioral, and cognitive instability. Safety and containment quickly merge into each other during crisis management, and likewise involve similar general treatment modules supplemented with specific interventions consisting of scheduled and/or PRN medications. During this phase, the treatment alliance and motivation/commitment to change begin to develop, and a consistent treatment process starts to form between the patient and their clinician.
*Phase 3 of Change: Control and regulation.* The primary goal is to increase self-regulation of maladaptive cognitions, affects, impulses, and behaviors. During this phase, general change mechanisms continue to build motivation/commitment to change and improve meta-cognition. These are typically supplemented with specific cognitive-behavioral techniques that promote self-regulation in these areas. Medications have limited utility in treating personality pathology after the containment phase (Khalifa et al., 2010; Lieb, Völlm, Rücker, Timmer, & Stoffers, 2010).
*Phase 4 of Change: Exploration and change.* The primary goal is to increase exploration and modulation of the maladaptive cognitive–affective structures related to interpersonal problems. General change mechanisms can be supplemented with cognitive-behavioral methods and psychodynamic techniques that promote awareness of maladaptive interpersonal schemas and their origins, and begin to restructure them.
*Phase 5 of Change: Integration and synthesis.* The primary goal is to construct an adaptive sense of self and resolution of interpersonal problems. The transition to this phase is typically seamless because the process of exploring and resolving interpersonal problems is intimately tied up with discussions with the patient about issues of identity and self-direction. General treatment modules are supplemented with specific interventions that promote restructuring of maladaptive self-schemas, the formation of an adaptive self-narrative and coherent sense of self, and the construction of a “personal niche” whereby the patient can meaningfully engage in work, love, and play.

Therefore, as we can see, general treatment modules are used throughout all phases of change, and the clinician supplements them with specific treatment modules tailored to target the current domain of dysfunction. Thus, Phases 1 and 2 are concerned primarily with treating acute symptoms, Phase 3 with treating difficulties in regulation and modulation, Phase 4 with treating interpersonal problems, and Phase 5 with treating self problems.

With this basic outline of the IMT unpacked, the treatment implications of our Bayesian model of psychopathy become apparent and provide the basis for an *Integrated Modular Treatment for Psychopathy*. Recall that our model is centered on two hypotheses:

1.  Early adverse attachment experiences interact with genetic vulnerabilities to shape the development of a psychopathic patient’s core self-image (i.e., internal working model of the self) as worthless and shameful.
2.  The traits *lacks remorse* and *self-aggrandizing* are compensatory high-level prior beliefs (i.e., intermediate beliefs) that function as defense mechanisms, allowing the patient with psychopathy to eliminate or at least diminish (i.e., cope with) the influence of their negative core self-image on their conscious experience.

In other words, at the heart of our model is the idea that psychopathy is characterized by a core impairment in self functioning—namely, deep-seated feelings of worthlessness and shame arising from the internal working model of the self. This implies that general treatment modules likely play a primary role in the treatment of psychopathy, because the new experiences within treatment can provide a continuous corrective experience that challenges these core beliefs. Specifically, the core self-image of worthlessness, shame, and inadequacy are challenged by *consistent* and *regular* exposure to experiences with a clinician who (a) is supportive, empathic, and validating; (b) shows congruence/genuineness in the relationship; (c) is carefully attentive to and flexibly resolves ruptures in the treatment alliance; (d) has an attitude toward the patient consisting of collaboration, respect, care, and positive regard; and (e) models and reinforces, in a nonpunitive, nondevaluing, and nonshaming manner, appropriate boundaries and prosocial internal standards (Castonguay & Beutler, 2006; Clarkin et al., 2015; Critchfield & Benjamin, 2006; Livesley, 2003, 2005, 2007a, 2007b, 2012; Livesley et al., 2016; Mitchell, Tafrate, & Freeman, 2016; Tafrate & Mitchell, 2014). In this way, the general treatment modules of the IMT for psychopathy function analogously to the SFT concept of *limited re-parenting*, which “involves providing, within the appropriate boundaries of the therapy relationship, what patients needed but did not get from their parents as children” (Young et al., 2003, p. 177). These general treatment modules are likely counterintuitive to people who have never worked with antisocial individuals. However, as is well known in correctional rehabilitation, a “get tough,” “get real,” or confrontational attitude never works, and is likely iatrogenic, because it invariably leads to reactance, argumentativeness, disengagement and, eventually, shatters the working relationship and thus the possibility for treatment (Mitchell et al., 2016; Tafrate & Mitchell, 2014). In short, when it comes to the core of treatment in the IMT for psychopathy, patients with psychopathy are no different than any other patient with a PD: The same common factors are utilized and maximized throughout all phases of change.

The second treatment implication of our model is the use of specific treatment modules targeting the maladaptive intermediate beliefs (i.e., high-level prior beliefs) of *self-aggrandizing* and *lacks remorse* (i.e., the unique features of the patient). Once patients with psychopathic traits have transitioned into the control and regulation phase, the focus of specific interventions should turn to increasing self-regulation of these maladaptive thinking patterns. These thinking patterns are, in fact, well known in the risk assessment and correctional rehabilitation literatures. *Self-aggrandizing* and *lacks remorse* are two instances of a broader class of cognitions called *criminogenic thinking patterns*—one of the “Central Eight” strongest, most robust risk factors for violent and antisocial behavior (Andrews & Bonta, 2010a, 2010b; Bonta et al., 2014; Gendreau et al., 1996). Criminogenic thinking patterns are attitudes, values, and beliefs that facilitate violent/antisocial behavior, and are believed to operate at the level of intermediate beliefs (Mitchell et al., 2016; Seeler, Freeman, DiGiuseppe, & Mitchell, 2014), which is in keeping with our Bayesian model’s hypotheses (Figure 3). Moreover, given that criminogenic thinking patterns contribute significantly to the harm of others and a wide range of dysfunction in the patient’s life, almost all evidence-based correctional rehabilitation programs involve specific techniques to alter criminogenic thinking, and these techniques involve standard cognitive-behavioral methods (e.g., identifying automatic thoughts and their associated intermediate beliefs, increasing awareness of the link between thoughts, emotions, and behavior, Socratic questioning of beliefs, behavioral experiments, etc.; Lipsey et al., 2007). Therefore such techniques targeting criminogenic thinking patterns may be crucial modules for treating the maladaptive cognitions associated with *self-aggrandizing* and *lacks remorse* during Phase 3 of treatment. Such techniques would be part of an eclectic array of specific treatment modules used during this phase in order to increase the patient’s self-regulation of their maladaptive cognitions, affects, impulses, and behaviors.

In summary, our Bayesian model of *self-aggrandizing* and *lacks remorse* resonates closely with the IMT model of PD treatment. For this reason, we have provided a general overview of a novel Integrated Modular Treatment for Psychopathy which we believe, with further development, can provide a fruitful, evidence-based framework for translating the computational neuroscientific concepts discussed in this article into a treatment for psychopathy that can be evaluated for efficacy/tolerability and cost-effectiveness.

## CONCLUSION

In summary, the Bayesian model of *lacks remorse* and *self-aggrandizing* proposes that entrenched abnormalities in prior beliefs about the self and abnormalities in the encoding of precision result in the generation of maladaptive Bayesian inferences about the self. These inferences manifest as a grandiose self-image and remorseless disregard for the effect of one’s behavior on others. These inferences are generated because these pathogenic mechanisms serve a vital function for the patient: They defend against consciously experiencing the deep-seated feelings of worthlessness and shame arising from the patient’s internal working (or generative) model of the self, which is rooted in their early adverse attachment experiences. These traits may reflect the patient’s learned and reinforced defensive responses to their traumatic history of repeatedly being made to feel worthless and shameful in the eyes of others, particularly their attachment figures. Although there is a great need for more computational and neuroscientific research to elucidate the details, this Bayesian model of psychopathy is consistent with the major psychotherapeutic models in the field and with existing research on the neurobiology of psychopathy. Furthermore, we have provided a working quantitative simulation of this model, which means that, in principle, one could quantify the pathological prior beliefs underlying psychopathic traits (Schwartenbeck & Friston, 2016). Indeed, the quantification of psychopathology is one of the promises of computational psychiatry (King-Casas et al., 2008; Kishida, King-Casas, & Montague, 2010; Kishida & Montague, 2012; Moutoussis, Fearon et al., 2014; Moutoussis, Trujillo-Barreto, El-Deredy, Dolan, & Friston, 2014; Ray, King-Casas, Montague, & Dayan, 2009). Finally, we provided a preliminary description of the treatment implications of our model through a general overview of a novel Integrated Modular Treatment for Psychopathy. Though much work still needs to be done, we hope that this article lays a foundation for integrating cognitive and psychodynamic approaches with well-established computational frameworks in neuroscience in the hope of bringing the field closer to understanding the etiology, and therefore treatment, of psychopathy.

## AUTHOR CONTRIBUTIONS

AP, KF, and NB contributed to the conceptualization of the Bayesian model of psychopathy. KF and TP performed the quantitative simulations. AP developed the treatment implications of the Bayesian model of psychopathy. AP and KF wrote the original draft. AP, KF, NB, and TP reviewed and edited the article.

## FUNDING INFORMATION

AP, KF, NB, and TP received no financial support for this project and have no conflicts of interest to declare. KF is funded by a Wellcome Trust Principal Research Fellowship (088130/Z/09/Z). TP is supported by the Rosetrees Trust (award 173346). The UCL Open Access Team kindly covered the open access fees.

## Notes

^1^ The P&PDWG proposed this model of personality disorders for inclusion in the *DSM–5*. The P&PDWG’s model was approved by the *DSM–5* Task Force; however, in the end, the Board of Trustees of the American Psychiatric Association rejected this model for placement in the main section of the *DSM–5* (Section II), and it was placed in Section III (“Emerging Measures and Models”) under the title “Alternative DSM–5 Model for Personality Disorders” (AMPD). The *DSM–IV* categorical model was retained in Section II of the *DSM–5* virtually unchanged. The reasons why the Board of Trustees ultimately rejected the AMPD are complex and extend beyond the scope of this article. The reasons and history behind this decision are thoroughly detailed elsewhere (Morey et al., 2015; Skodol, 2014; Zachar, Krueger, & Kendler, 2016).^2^ Empirical priors are priors that occupy intermediate positions in hierarchical models. They are synonymous with intermediate posteriors.^3^ Strictly speaking, *β* only plays the role of a precision until it falls to a value of one half (at which point beliefs about state transitions are maximally imprecise at 50-50).^4^ The anatomical definition of the medial prefrontal cortex (mPFC) and its sub-regions varies between studies. In this article, the mPFC refers to the entire medial section of the PFC, including sections of the anterior cingulate cortex (ACC). The ventromedial PFC (vmPFC) encompasses the ventral portion of the mPFC, including the medial orbitofrontal cortex (mOFC), which corresponds to the medial sections of Brodmann area (BA) 11, 25, and lower section of BA 32. The dorsomedial PFC (dmPFC) encompasses the dorsal portion of the mPFC, which corresponds to the medial sections of BA 8, 9 and the upper section of BA 32. The rostromedial PFC (rmPFC) encompasses the most anterior pole of the mPFC (BA 10).^5^ Research suggests that the association between psychopathy and amygdala volumes is complex. Specifically, some amygdala nuclei are associated with volumetric reductions in patients with psychopathy, whereas other nuclei are associated with enlargement (Boccardi et al., 2011; Yang et al., 2010; Yang, Raine, Narr et al., 2009).^6^ The default mode network (DMN) consists of a network of regions that show increased, synchronized activity during states when the brain is at rest compared to active, externally directed tasks (Greicius, Supekar, Menon, & Dougherty, 2009; Gusnard & Raichle, 2001; Raichle, 2015; Shulman et al., 1997). While there is still debate over the precise function of the DMN, it appears to support *internal mentation*, i.e., stimulus-independent, task-unrelated thought (Andrews-Hanna, 2012). The DMN regions include the dorsomedial prefrontal cortex (dmPFC), ventromedial prefrontal cortex, (vmPFC), posterior cingulate cortex (PCC), precuneus (pC), inferior parietal lobule (IPL), temporal parietal junction (TPJ), lateral temporal cortex (LTC), temporal pole (TP), retrosplenial cortex (Rsp), entorhinal cortex (ERC), parahippocampal cortex (PHC), and hippocampus (Andrews-Hanna, 2012; Andrews-Hanna, Reidler, Sepulcre, Poulin, & Buckner, 2010; Buckner, Andrews-Hanna, & Schacter, 2008).^7^ When using such intensive specific interventions, it is recommended that (1) they be the *least onerous* and *least restrictive*, (2) duration is determined using a *structured evidence-based assessment of risk*, with the view to make them as brief as possible, (3) that *due process* be in place to protect the patient’s rights, and (4) that the interventions be implemented, to the greatest extent possible, so as to maximize the patient’s perception of *procedural justice/fairness* and minimize their *perception of coercion*.
